# Engineering Human Cardiac Muscle Patch Constructs for Prevention of Post-infarction LV Remodeling

**DOI:** 10.3389/fcvm.2021.621781

**Published:** 2021-02-26

**Authors:** Lu Wang, Vahid Serpooshan, Jianyi Zhang

**Affiliations:** ^1^Department of Biomedical Engineering, School of Medicine and School of Engineering, University of Alabama at Birmingham, Birmingham, AL, United States; ^2^Department of Biomedical Engineering, Emory University School of Medicine and Georgia Institute of Technology, Atlanta, GA, United States; ^3^Department of Pediatrics, Emory University School of Medicine, Atlanta, GA, United States; ^4^Children's Healthcare of Atlanta, Atlanta, GA, United States

**Keywords:** tissue engineering, cardiac patch, myocardium, heart failure, myocardial infarction, regenerative medicine, cardiac regeneration and remodeling

## Abstract

Tissue engineering combines principles of engineering and biology to generate living tissue equivalents for drug testing, disease modeling, and regenerative medicine. As techniques for reprogramming human somatic cells into induced pluripotent stem cells (iPSCs) and subsequently differentiating them into cardiomyocytes and other cardiac cells have become increasingly efficient, progress toward the development of engineered human cardiac muscle patch (hCMP) and heart tissue analogs has accelerated. A few pilot clinical studies in patients with post-infarction LV remodeling have been already approved. Conventional methods for hCMP fabrication include suspending cells within scaffolds, consisting of biocompatible materials, or growing two-dimensional sheets that can be stacked to form multilayered constructs. More recently, advanced technologies, such as micropatterning and three-dimensional bioprinting, have enabled fabrication of hCMP architectures at unprecedented spatiotemporal resolution. However, the studies working on various hCMP-based strategies for *in vivo* tissue repair face several major obstacles, including the inadequate scalability for clinical applications, poor integration and engraftment rate, and the lack of functional vasculature. Here, we review many of the recent advancements and key concerns in cardiac tissue engineering, focusing primarily on the production of hCMPs at clinical/industrial scales that are suitable for administration to patients with myocardial disease. The wide variety of cardiac cell types and sources that are applicable to hCMP biomanufacturing are elaborated. Finally, some of the key challenges remaining in the field and potential future directions to address these obstacles are discussed.

## Introduction

Despite advancements in preventive medicine, cardiovascular disease (CVD) remains a leading cause of morbidity and mortality worldwide (1, 2) with estimated 17.9 million people died of cardiovascular disease in 2016, accounting for 31% of all deaths globally (3). The molecular and cellular basis for progressive heart failure is the result of the inability of damaged and apoptotic myocytes to be replaced. The regenerative capacity of mammalian hearts declines rapidly after birth, and <1% of cardiomyocytes (CMs) in the hearts of adult humans are replaced each year (4); thus, myocardial injury leads to adverse cardiac remodeling and fibrosis as the injured myocardium is replaced by fibrotic scar tissue (5, 6). The only available procedure for end-stage patients is whole-heart transplantation which is restricted by an inadequate supply of donors. Therefore, alternative strategies for limiting post-injury cardiac remodeling and remuscularizing the damaged myocardium are urgently needed (7, 8). Administration of exogenous stem cells was initially considered a promising approach (9). The cells were expected to differentiate into CMs after transplantation (10, 11); however, the results from subsequent preclinical and clinical studies indicated that the benefits were only marginal and likely attributable to the cells' paracrine activity, because the proportion of cells that were retained and survived at the site of injury (i.e., the engraftment rate) was drastically low (12, 13).

**Figure 1:**
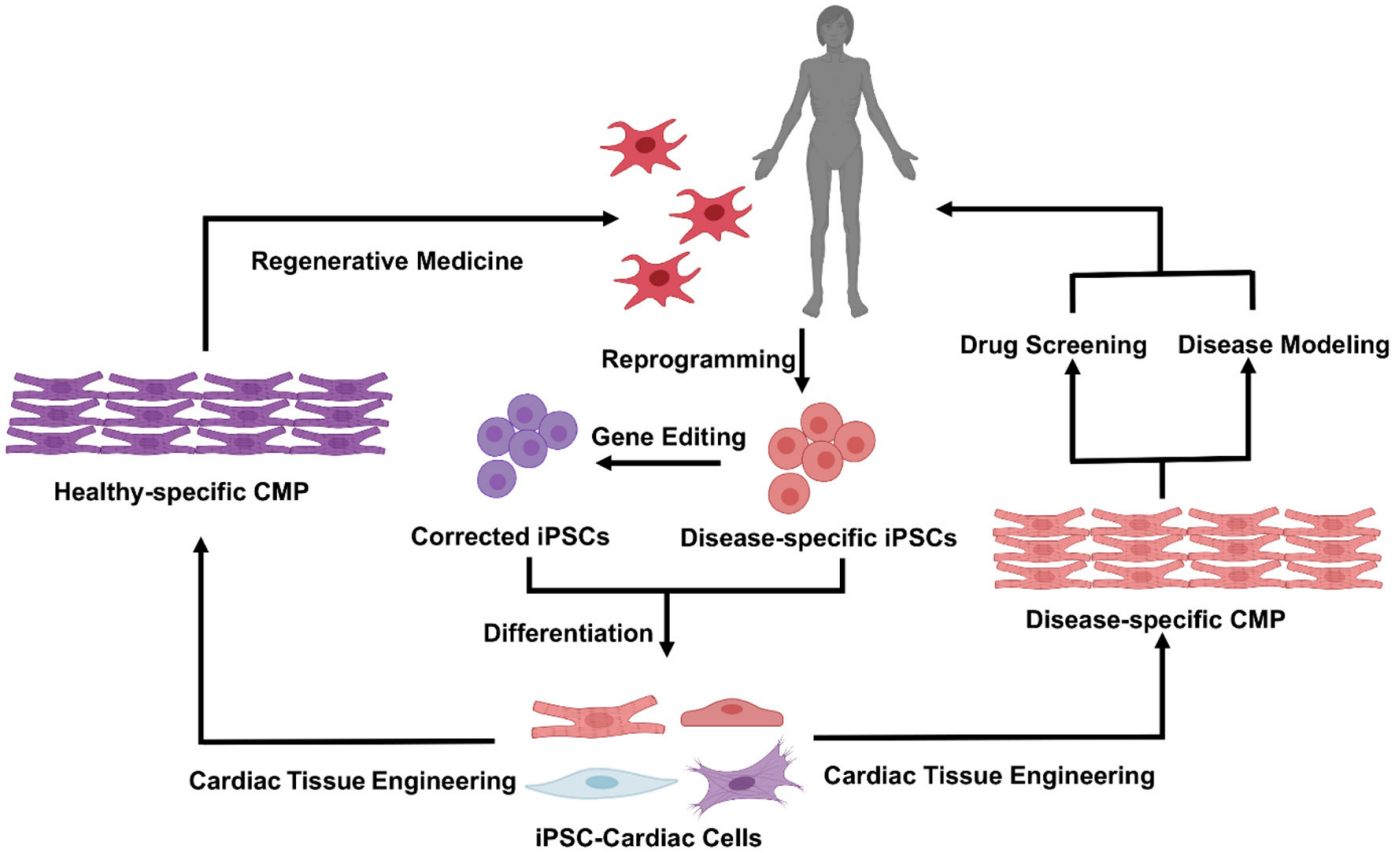
Cardiac tissue engineering and its applications. Patient specific iPSC can be derived by reprogramming of somatic cells from the patient, in healthy vs. diseased states, and used to generate a variety of functional cardiovascular cells. Incorporation of cells within specifically tuned 3D biomaterial systems will enable fabrication of the human cardiac muscle patch (hCMP) that could be used either in a variety of *in vitro* applications [drug screening and disease modeling **(Right)**], or as cardiac patch for *in vivo* regenerative therapies **(Left)**.

Tissue engineering combines the principles of engineering and life sciences to better understand the structure-function relationships in normal and pathological tissues and to generate living tissue equivalents for drug testing (14, 15), disease modeling (16–18), and regenerative medicine [(6, 19, 20); Figure 1]. In the cardiovascular field, the first proof-of-concept study was conducted in 1997, when embryonic chick CMs were suspended in collagen solution, and the mixture subsequently solidified and contracted coherently between two glass tubes (21). With the emergence of techniques for reprogramming human somatic cells into induced pluripotent stem cells (iPSCs) and differentiating them into CMs (22) and other cardiac cells, the field has progressed to the development of engineered human cardiac muscle patch (hCMP) constructs (6). These engineered patch constructs are often associated with higher rates of engraftment and appear to support the injured myocardium more effectively than transplanted cells (2, 23). Nevertheless, vascularization of the hCMP (either during manufacture or via the infiltration of native vessels after transplantation) is not extensive and efficient enough to support the high metabolic demand of the heart (13, 24). Consequently, the thickness/dimensions of most hCMPs are limited to just a few hundreds of micrometers (13, 25). In this review, we discuss many of the most recent advancements in cardiac tissue engineering, with a primary focus on techniques for generating thicker and more integrative hCMP systems.

### Cell Types and Sources for hCMP Fabrication

**Figure 2:**
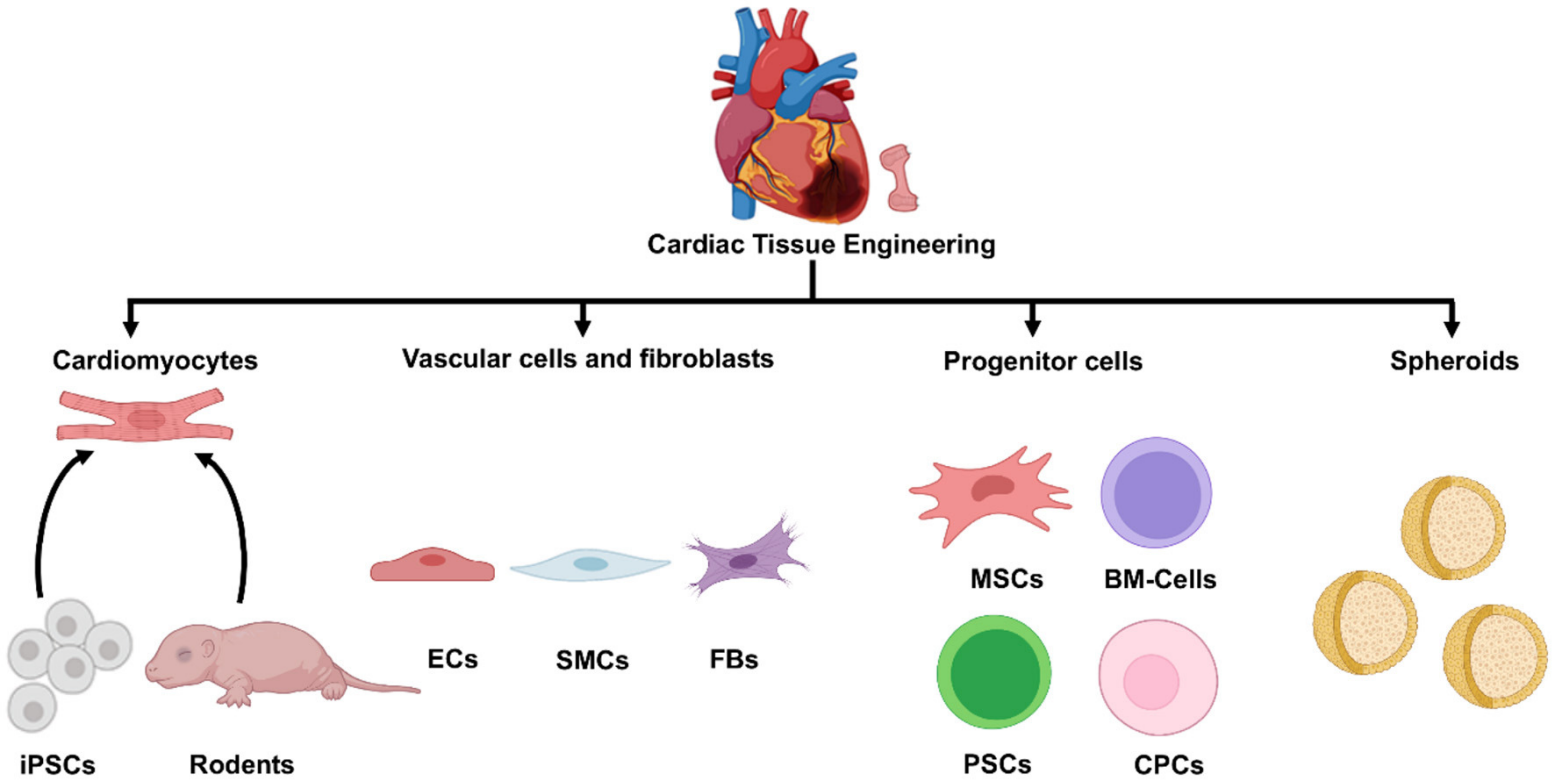
Cell sources for cardiac tissue engineering. A variety of cell types can be used in tissue engineered cardiac constructs, including cardiomyocytes derived from iPSCs or isolated from rodent hearts, cardiac vascular cells and fibroblasts, different progenitor and stem cells, and various spheroids. iPSC, induced pluripotent cells; ECs, endothelial cells; SMCs, smooth muscle cells; FBs, fibroblasts; MSCs, mesenchymal stem cells; BM-cells, bone marrow-cells; PSCs, pluripotent stem cells; CPCs, cardiac progenitor cells.

CMs are the fundamental contractile units of the myocardium and occupy 70–85% of myocardial volume in adult mammals (26); thus, many investigations of cell therapy have been conducted with CMs alone, either as dissociated cells or contiguous sheets (27, 28). However, hCMPs are designed to comprehensively recapitulate the physical structure and signaling pathways present in native heart tissue (13, 29) and, therefore, are typically composed of multiple cardiac cell types, including CMs, endothelial cells (ECs), smooth muscle cells (SMCs), and cardiac fibroblasts (6, 26, 30). Other cell types or clusters of multiple cell types (e.g., progenitor cells and spheroids) have also been incorporated into cardiac patches and evaluated in preclinical models of myocardial injury (Figure 2).

#### Cardiomyocytes (CMs)

Healthy adult human CMs were largely unavailable for early hCMP studies due to scarcity of healthy heart donors and their non-proliferative phenotype. Thus, most of what we have explored and learned about the structural and functional properties of engineered cardiac tissues was initially investigated in experiments using primary rodent CMs (31). Pioneering work with fetal CMs from 15-day-old mouse embryos demonstrated that the cells were engrafted and survived after administration into the mouse hearts (32). Rhythmically contracting hydrogels were generated by plating neonatal rat heart cells on collagen, which enabled researchers to study how factors, such as cell density and collagen concentration, influenced contractile activity (33) and to demonstrate that these cellular constructs generated electrocardiography (ECG)-like potentials (34). Subsequent studies confirmed that patches composed of fetal rat ventricular cells and gelatin could survive and continue to contract when implanted subcutaneously in the adult rat legs. The engineered graft formed junctions with native heart cells when delivered to the scarred region of cryoinjured hearts, but whether the treatment could improve cardiac function remained uncertain (35). Patches consisting of fetal rat cardiac cells suspended in an alginate scaffold were among the first to preserve measures of cardiac function and impede adverse cardiac remodeling when administered to infarcted rat hearts (36).

Embryonic stem cells (ESCs) and iPSCs are the most readily available sources of human-lineage CMs, because they can proliferate indefinitely and be differentiated into cells of different lineages (37). The first human vascularized, contracting hCMP was generated by combining CMs and ECs derived from human ESCs with mouse embryonic fibroblasts in porous sponges composed of 50% poly-l-lactic acid (PLLA) and 50% polylactic-glycolic acid (PLGA) (38). Experiments in both rodent (19, 39, 40) and swine (23) models of myocardial injury suggest that iPSC-based hCMPs are associated with higher levels of cell survival and engraftment than those associated with iPSC-based cell injection, and that the cells' paracrine activity can be modestly beneficial. Both ESC- and iPSC-derived CMs are, however, structurally and functionally more similar to fetal or neonatal cells than to the adult mature CMs. This could hinder downstream applications of hCMPs composed of immature PSC-CMs. The electrical immaturity of PSC-CMs may induce ventricular arrhythmias as demonstrated in non-human primate models, raising a safety concern for clinical translation (41, 42). Therefore, various methods for improving stem cell-derived CM maturity before transplantation, including electrical stimulation, and treatment with neurohormonal factors, have been frequent topics of research (43–46). hCMP maturation can be influenced by the purity of the initial CM population, as evidenced by increases in conduction velocity and contractile force (44). Further, mechanical conditioning of cardiac patches has been shown effective in maturation and functional improvement of encapsulated CMs. For instance, hCMPs consisting of a collagen matrix and CMs differentiated from mouse ESCs, more closely resembled native myocardial tissue after undergoing 7 days of *in vitro* cyclic stretching (47).

#### Cardiac Vascular Cells and Fibroblasts

Cardiac ECs and SMCs not only promote the vascularization and survival of transplanted hCMPs but are also key mediators of the signaling mechanisms that regulate CM activity (23, 48, 49). Cardiac fibroblasts can further improve CM survival by remodeling the extracellular matrix (ECM) and releasing cytoprotective paracrine factors (6, 9, 50–52). Except for providing a reliable source of CMs, hiPSCs demonstrate great potential in offering vascular cells and fibroblasts. Robust protocols for differentiating hiPSCs to ECs (53), SMCs (50), and cardiac fibroblasts (54, 55) have been established. The inclusion of ECs and stromal cells, combined with uniaxial mechanical stimulation during manufacturing, has been shown to improve the hCMP maturity (56). However, early protocols for hiPSC-EC differentiation are not efficient and the phenotype of generated hiPSC-ECs remain rather unstable. Refined EC differentiation protocols, utilizing spatiotemporal 3D environments, have prolonged the maintenance of EC phenotype to up to 4 weeks (57). With deeper insights into the signaling pathways affecting cardiac cell differentiation, novel protocols for EC and SMC differentiation have yielded efficiencies exceeding 80% within 6-day time periods (58). Great efforts have also been devoted to deriving arterial EC from hiPSC, which demonstrated arterial-specific functional characteristics unlike generic ECs, thus offering reliable cell sources for hCMP (59). Cardiac fibroblasts have been successfully differentiated from hPSCs, which resembled native cardiac fibroblasts in morphology, gene expression, and proliferation. Notably, hPSC-derived cardiac fibroblasts could generate three-dimensional (3D) ECM scaffolds and their co-culture with hPSC-CMs could increase action potential propagation rate compared to co-culture with dermal fibroblasts (60). Despite development of refined protocols for generation of vascular cells and fibroblasts of arterial lineage, the optimal combination and proportion of cell types for recapitulating the complex 3D environment of native heart tissue continues to be an active area of research (52, 61–64).

#### Pluripotent Stem Cells

Pluripotent Stem Cells (PSCs), including ESCs and iPSCs, have revolutionized the field of cardiac tissue engineering by providing a platform of unlimited numbers and types of cells due to their ability to self-renew indefinitely and the plasticity to differentiate into any type of cells (6, 45). ESCs were initially derived from inner cell mass of developing blastocyst (65), thus raising ethical issues due to destruction of fertilized human embryos. ESCs could also be collected via parthenogenesis which alleviates the ethical issues (66). In contrast, iPSCs were initially reprogrammed from somatic cells via overexpression of four transcription factors, Oct4 (octamer-binding transcription factor 4), Sox2 (sex determining region Y-box 2), Klf4 (KLF family of transcription factor 4), and Myc (67). Different combinations of transcription factors, such as Oct4, Sox2, Nanog, and Lin28 (68) or only Oct4 and Sox2 (69) can be also used to generate iPSCs. Despite the controversy on immunogenicity of autologous iPSCs (70–73), devoid of ethical issues qualifies them as one of the most promising cell sources for cardiac tissue engineering.

PSCs have revolutionized biomedicine by providing robust platforms for regenerative medicine, drug screening, and disease modeling, all requiring reproducible and efficient protocols for differentiating PSCs to CMs. Initially, contractile CMs were spontaneously differentiated from hESCs in 3D embryoid bodies (EBs) with low efficiencies (74–76). These early protocols suffered from line-to-line variability, inclusion of undefined components, and heterogeneous EB sizes (77, 78). To advance the yield and purity of CM differentiation protocols, in-depth knowledge of the pathways involved in embryonic heart developments was utilized to optimize the process. CM differentiation stages typically involve early mesendoderm priming, cardiac progenitor specification, and differentiation into CM subtypes such as ventricular and atrial like phenotypes (79–81). Modulation of TGFβ signaling superfamily via serial application of activin A and bone morphogenetic protein 4 (BMP4) yielded >30% CMs (82); manipulation of Activin/Nodal and BMP4 signaling pathways could efficiently induce cardiac mesoderm, resulting >60% CMs in mouse PSCs and >50% in human PSCs (83). Robust CM differentiation protocols have been developed by mimicking biphasic pattern of WNT pathway with chemical compounds. Temporal modulating Wnt pathway via GSK inhibitor CHIR99021 and Wnt inhibitor IWP2 generated 80–98% CMs (84). Suspension culture in stirred tank bioreactors was utilized to upscale hPSC expansion and lineage differentiation (85, 86). Efforts have been made to develop suspension culture based hPSC-CM production with good manufacturing practice standards (79, 87). Pioneering works of hCMP with PSC-CMs have laid foundation for optimization of manufacturing methods and demonstrated beneficial efficacy after transplantation into animal models with higher engraftment and survival rate than those associated with iPSC-based cell injection (38, 47, 56). Notably, hCMP fabrication typically incorporates PSC-derived cardiac cells with refined protocols to differentiate PSC into ECs, SMCs, and fibroblasts which benefit hCMPs via pre-vascularization and secretion of ECM proteins (50, 52).

#### Progenitor Cells

The results from early clinical trials suggested that the modest benefits associated with transplanted bone marrow-derived cells and cardiac progenitor cells (CPCs) occurred via the cells' paracrine activity, rather than by directly repairing the damaged heart tissue (30). Nevertheless, bone marrow mesenchymal stem cells (MSCs) have been suspended in 3D hydrogels and tested in a rat model of myocardial infarction (MI) (88). The development of a program for generating human ESC-derived CPCs under Good Manufacturing Practice (GMP) conditions (89) led to the first clinical trial of hESC-CPCs, in which the cells were administered by suspending them in fibrin and suturing the patch to the surface of the infarcted heart. Heart function improved symptomatically and no safety concerns were observed 3 months after transplantation (90). Further research was conducted to investigate the efficacy of ESC-derived cardiovascular progenitors administered in a fibrin patch in six patients with severe ischemic left ventricular dysfunction. Results demonstrated uneventful recoveries with no complications such as tumor formation, arrhythmias or alloimmunization (91). These trials demonstrated feasibility and safety of clinical translation of hESC-derived cardiovascular progenitors, paving the way for further efforts of efficacy studies. Recently, CPCs have shown promise as a highly reproductive cell source in additive cardiac tissue manufacturing, as an alternative to the non-proliferative, mature CMs, to create highly cellularized function hCMPs (92–94).

#### Spheroids

During the iPSC differentiation process, the cells tend to aggregate into spheroids containing multiple cell types, which can be used for high-throughput screening of heterocellular interactions and drug testing (6, 9). Spheroids could also enhance differentiation and promote maturation, with CMs comprising 80–100% of the cells in microtissues, while refined CM differentiation protocols in monolayer cultures could yield 80–98% CMs (84, 95). Furthermore, spheroids containing proportions of iPSC-derived CMs, ECs, and fibroblasts somewhat recapitulate the morphological and physiological properties of human myocardium, as well as the response to treatment with pharmacological agents, which suggests that spheroids could provide a valuable platform for *in vitro* disease modeling (96, 97). Measurements of engraftment and survival also appear to be greater when cells are administered in the form of spheroids, rather than disaggregated cellular suspensions (98, 99). In another study, engrafted spheroids composed of CPCs expressing the ISL1-LIM-homeodomain transcription factor differentiated into CMs and ECs and contributed to the formation of new blood vessels in infarcted mouse hearts (100). The maturation of iPSC-CM spheroids can be increased via mechanical and electrical stimulation (101, 102), but measurements of isometric force and electrical conduction are hindered by the lack of a functional architecture (9). The use of cardiac spheroids in advanced biomanufacturing procedures, such as bioprinting, has recently attracted increasing attention, as they can provide improved printed cell viability, fusion, and function to fabricate large-scale cardiovascular constructs (103, 104).

### Advanced Cardiac Tissue Manufacturing Strategies

**Figure 3:**
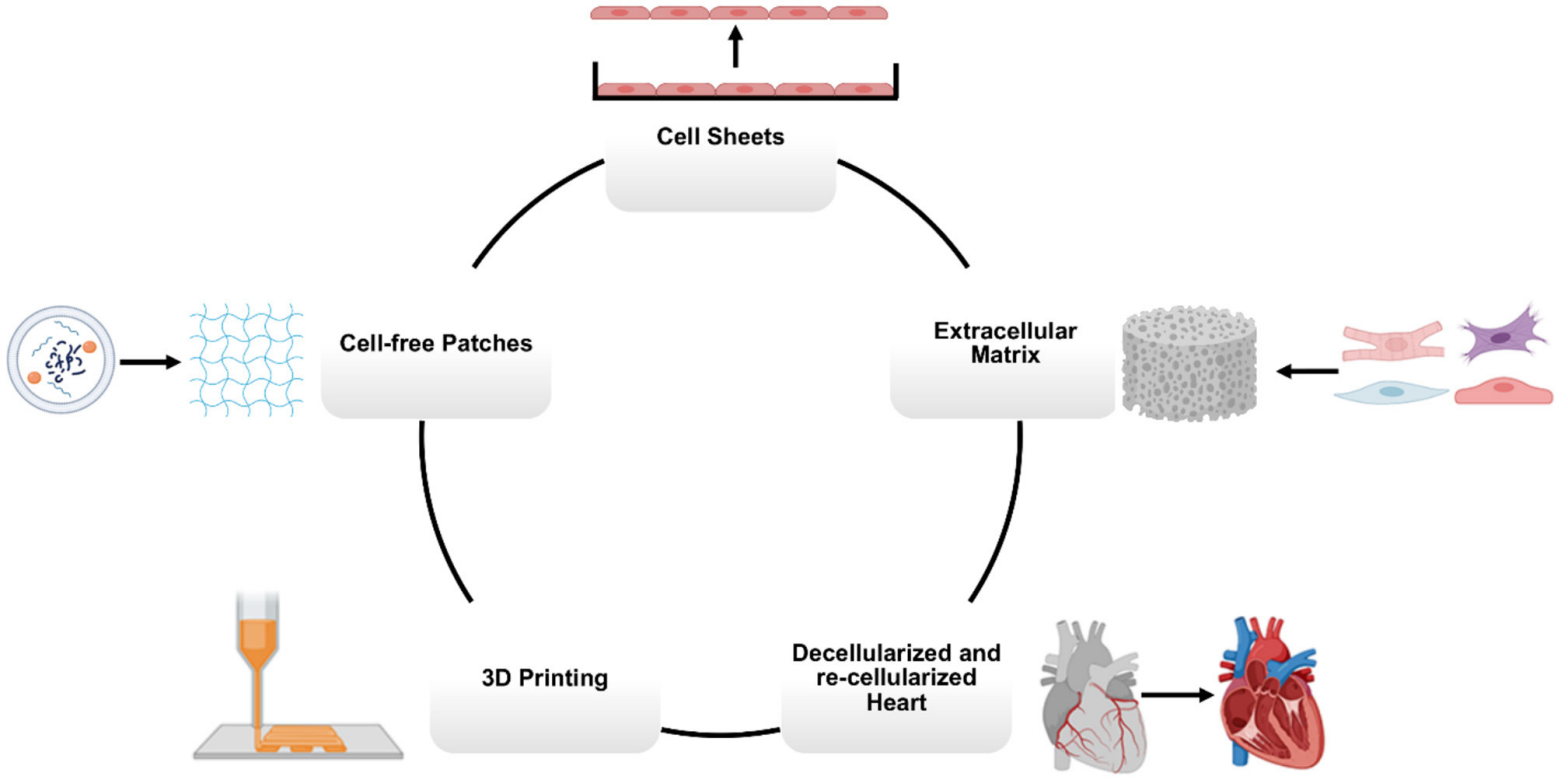
Main manufacturing methods for cardiovascular tissue engineering. Human cardiac muscle patch (hCMP) constructs can be fabricated using a variety of bioengineering methods, including cell sheets, scaffolds, decellularized heart tissues, 3D (bio)printing, and cell-free patches.

Conventional methods for manufacturing hCMPs include generating contiguous sheets of cardiac cells (mainly CMs) or suspending cells of a variety of types in scaffolds of biocompatible material (94, 105–108). More recently, the emergence of 3D printing technologies, combined with cell-containing “bioinks” and computer-aided design (CAD), has enabled researchers to define the architecture of hCMPs with previously unattainable precision (Figure 3).

#### Cell Sheet Approaches to Fabricate hCMPs

Cell sheets are typically produced by culturing cells on dishes coated with a temperature-sensitive polymer, such as poly(N-isopropyl acrylamide) (PIPAAm), which releases the attached cells when the temperature is reduced from 37 to 32°C, thereby maintaining the ECM and intercellular connections produced during the culture period (109). Since cell sheets lack the exogenous/synthetic scaffold, concerns regarding the potential immunogenicity of the scaffold material are abolished. The sheets can be stacked to generate 3D hCMPs that contract spontaneously (110). Furthermore, the cells of adjacent layers form connections that facilitate communication between layers, including gap junctions, which are required for electrical coupling (111). Although, constructs composed of more than four cellular layers are typically resistant to vascularization, the vascularity of fabricated cell sheets can be improved by the inclusion of stromal cells or omentum during manufacture (112, 113). Further, CM survival and contractile function were significantly improved when iPSC-CM sheets were covered with an omentum flap after transplantation into infarcted pig hearts (114–116). However, transplanted cell sheets tend to remain electromechanically isolated from the native myocardium, which suggests that the observed benefits likely occur primarily through the activation of paracrine mechanisms (117). Clinical trials utilizing cell sheets of skeletal myoblast demonstrated safety and feasibility with no complications, paving the way for further therapeutic efficacy studies (118, 119). Notably, the Japanese health ministry recently approved the use of iPSC-derived CM sheets in a small study of patients with heart disease, which represents only the second clinical application of iPSC-derived cells (115, 120).

#### Extracellular Matrix

The biomaterials used in scaffold-based hCMPs (121) are designed to mimic the native cardiac ECM which consists of a highly specialized, 3D network of structural (e.g., collagen) and non-structural (e.g., glycoproteins, proteoglycans, and glycosaminoglycans) proteins that support cardiac function (122, 123). The patch biomaterial is aimed to provide the architecture for cell attachment and cell-cell or cell-matrix interactions (122, 124) and facilitate the coordinated transmission of electrical and mechanical signals (9). Therefore, several design criteria for scaffold biomaterials have been established. *Biocompatibility* of materials is critical to minimize immunogenicity and cytotoxicity, while promoting cell attachment, differentiation and proliferation (30, 125). *Biodegradability* is another important aspect of scaffolding biomaterials, allowing the material to degrade at an appropriate rate while new ECM proteins are being synthesized (106, 126). *Biomechanical and biophysical properties* of materials such as stiffness, elasticity, and porosity enable effective electromechanical coupling of cardiac scaffolds and facilitate mass transport within the constructs (9, 94, 127, 128). Toward that goal, strategies utilizing natural hydrogels (e.g., collagen, fibrin, and Matrigel), synthetic polymers (e.g., polyglycolic acid, polycaprolactone, polylactic acid, and poly-D, L-lactic-co-glycolic acid), or nanofibrous materials as scaffolds have been attempted (9, 129–135). Natural materials have the advantages of promoting cell attachment and viability without cytotoxicity. Nevertheless, physical properties of natural materials could vary from batch to batch and source to source. Immunogenicity is also a concern for xeno-transplantation. Synthetic materials could be designed to precisely control mechanical properties and degradation rate, thus inducing minimal immunogenicity. However, the novel composition of synthetic materials often struggles to fully support cell adhesion and survival. Various scaffold architectures and geometries that have been tested include cylindrical constructs, which typically contain only small numbers of cells, and therefore, are usually scaled to much larger hCMPs at clinically relevant dimensions (2 cm × 4 cm × 1.25 mm) (23). Other structures used are various rodent-sized ventricular organoids (9).

#### Decellularized Heart Tissues

The role of the ECM in cardiac differentiation, organization, and vascularization is well-established by many studies with decellularized heart tissues (136). The ECM from decellularized human ventricles is shown to induce CM gene expression in human CPCs and MSCs. Further, human umbilical vein ECs formed endocardial and vascular linings in these constructs, and fully differentiated human CMs aggregated into muscular bundles with mature calcium dynamics and electrical coupling (137). Whole rat hearts have been also decellularized, seeded with cardiac and endothelial cells, and perfused in bioreactors, demonstrating macroscopic contractions after only 4 days of culture. By day 8 in culture, the recellularized hearts displayed pumping function in response to electrical stimulation, equivalent to about 2% of adult rat hearts (138). Even in the absence of seeded cells, application of a decellularized porcine myocardial tissue resulted in significant improvements in cardiac functional parameters when evaluated in a rat model of MI, and the patch was vascularized by cells that had migrated from the native tissues (139).

#### 3D Bioprinting—Additive Biomanufacturing

3D bioprinting technologies utilize CAD modeling to guide the assembly of living cells and other biological materials into large-scale tissue and organ analogs with precisely controlled, native-like architectures (92, 140–143). In an early work, tissue printing technology was utilized to generate a construct composed of human cardiac-derived CPCs and alginate. The printed construct remained committed to cardiac lineage with high viability after culturing for 7 days (144). In subsequent studies, human CM progenitor cells were bioprinted into a matrix of hyaluronic acid and gelatin to form a patch with six perpendicularly printed layers and a total surface area of 4 cm^2^. When evaluated in a murine MI model, the patch improved measures of cardiac function and prevented remodeling. The expression of cardiac and vascular differentiation markers increased during the 4-week follow-up period (145). Scaffold-free bioprinted hCMPs have been also generated by loading spheroids, one-by-one, onto an array of needles, allowing them to fuse, and then removing the hCMP and culturing it until the needle holes were filled in with surrounding tissue. The construct remained engrafted and displayed evidence of vascularization 1 week after implantation into the infarcted rat hearts (104). A more recent, customized device has been developed that can load an entire layer of spheroids onto the needle array simultaneously, which will substantially reduce the time required to print larger engineered constructs (146).

The resolution of traditional bioprinting techniques cannot accommodate the structural details that facilitate interactions with individual cells. To address this limitation, a more advanced technique, multiphoton-excited (MPE) 3D bioprinting, has emerged, allowing the control of the architecture of photoactive polymers to resolutions of <1 μm and, consequently, reproducing the structural features of the ECM with high fidelity (147). MPE 3D-printed hCMPs composed of iPSC-derived CMs, ECs, and SMCs in a photoactive gelatin scaffold began generating calcium transients and beating synchronously within 1 day post-manufacturing. The printed patches were associated with significant improvements in cardiac function (left ventricular ejection fraction and fractional shortening), infarct size, apoptosis, vascularity, and cell proliferation when tested in mice with surgically induced MI (62). Photoactivated 3D bioprinting has also been conducted with a bioink containing both ECM proteins and hiPSCs to generate two-chambered structures with both inlet and outlet vessels. The hiPSCs proliferated and differentiated into mature cardiac cells *in situ* to form a living pump with contiguous walls of human cardiac muscle that mimicked the chambers and large vessels of a native heart (148).

#### Cell-Free Cardiac Patches

Cell- and tissue-based treatments will likely require specialized methods of storage and transportation to maintain cell viability (149). Since most of the benefits associated with transplanted cells and engineered tissues appear to evolve from the paracrine factors produced by the cells, rather than the cells themselves (150), strategies that exploit the regenerative activity of these paracrine factors may be more easily translated to the clinics. The three primary categories of secreted, biologically active cellular products are growth factors (typically proteins that function as signaling molecules), non-coding RNA (short, single-stranded oligonucleotides that regulate gene expression), and extracellular vesicles of endosomal origin (151). Artificial cardiac patches have been manufactured by encapsulating the factors secreted by cardiac stromal cells in PLGA and then embedding the capsules in decellularized porcine myocardium. Applying these factor-laden patches to the infarcted hearts of both immunocompetent rats and pigs resulted in reduced scarring and improved cardiac function, without inducing an immune response (152). In other works, the controlled release of miRNAs from injected hydrogels has shown improvement in cardiac function in infarcted mouse hearts (153). Further, hydrogel patches containing the extracellular vesicles produced by human iPSC-CMs reduced infarct size and cardiac hypertrophy in a rat MI model (154). Collectively, these results suggest that the controlled release of paracrine factors from cell-free patches or injected biomaterials may be a feasible alternative approach to transplanted hCMPs for the treatment of myocardial disease.

### Increasing hCMP Thickness/Dimensions

**Figure 4:**
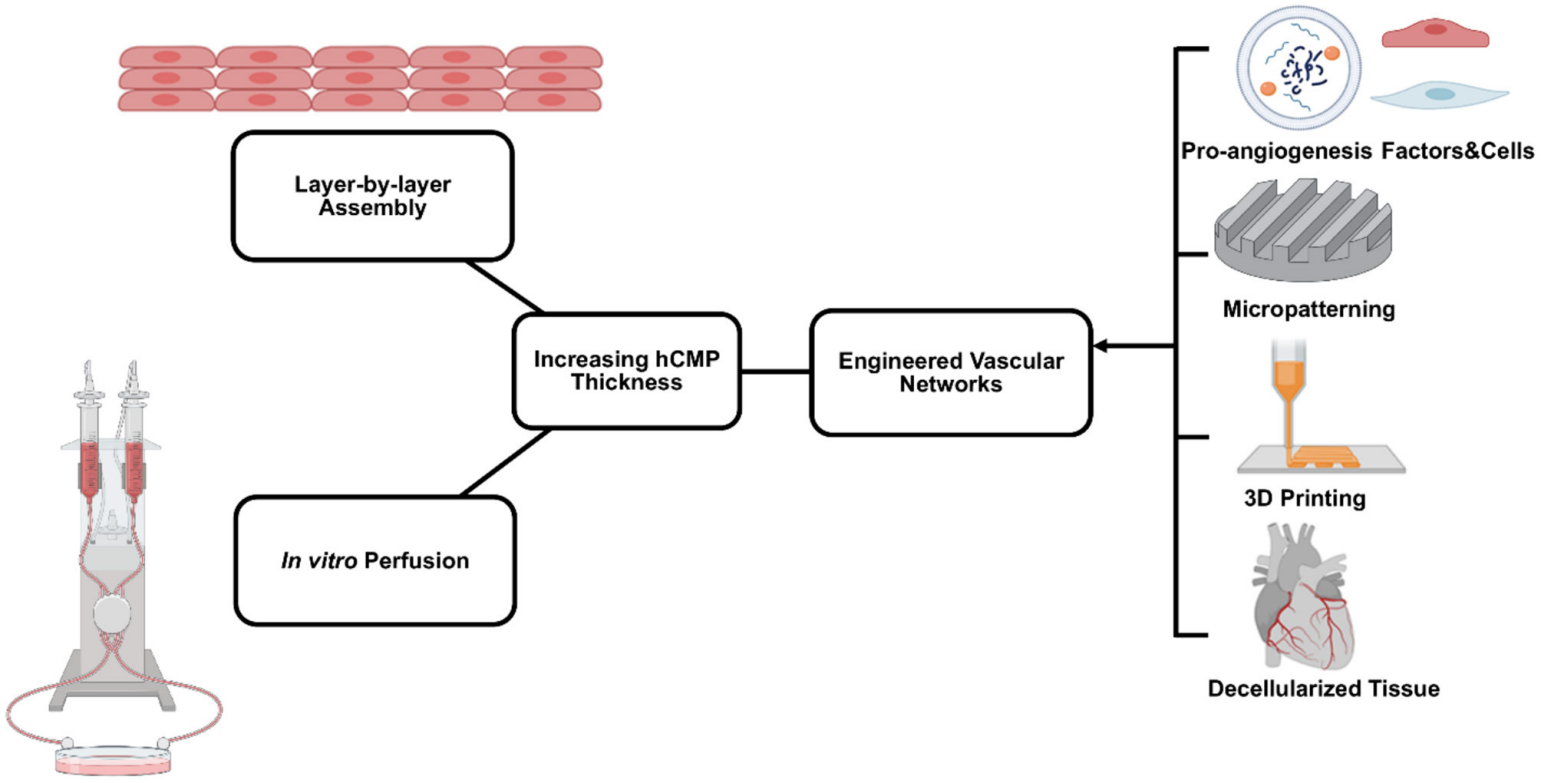
Primary bioengineering techniques to manufacture and maintain relatively thick human cardiac muscle patch (hCMP) constructs. A variety of methods are used, including layer-by-layer assembly, *in vitro* perfusion using various bioreactor systems, and engineered vascular networks.

Despite substantial improvements in the components and protocols used for hCMP manufacturing, few studies have been conducted with constructs of clinically relevant size. Even patches with comparatively large surface areas (e.g., 8 cm^2^) are relatively thin (1.25 mm) (23), with inability of direct perfusion limiting thickness of hCMP to 1–2 mm (155, 156). Thus, production methods continue to be optimized and enhanced for the development of larger and thicker hCMP constructs. The inability to engineer and maintain thick and viable hCMP jeopardizes the clinical applications due to their inability to mimic characteristics of native myocardium, such as generating adult-like forces and action potentials (157, 158). In practice, hCMP thickness is often limited by the diffusion of oxygen and nutrients from the vasculature after transplantation, which requires CMs to be within 100–200 μm distance from the capillaries (159). Diffusion can be facilitated by including molecular crystals (e.g., sucrose) in the matrix solution and leaching them out after the matrix solidifies to increase the porosity of the scaffold (160). However, hCMPs of clinically relevant thicknesses require formation of a dense internal vascular network that couples with the native circulation after transplantation (9, 157). Vascularization can be increased by including combinations of vascular and other cell types (ECs, SMCs, and/or fibroblasts) (23, 62, 161) during manufacturing and/or via the nanoparticle-mediated (151) extended release of pro-angiogenic factors [e.g., vascular endothelial growth factor (VEGF), fibroblast growth factor (FGF), and the Wnt activator CHIR99021; (162, 163)], which can promote infiltration of the native circulatory system. Further, the spatial orientation of the vascular network can be controlled with more technologically advanced fabrication methods (e.g., micropatterning and 3D bioprinting) to enhance the mass transport and perfusion [(164); Figure 4].

#### Layer-By-Layer Assembly

In theory, the layer-by-layer assembly technique enables hCMPs of any desired thickness to be generated simply by stacking the required number of individual CM sheets. However, since the individual layers of multilayered hCMPs are grown in isolation before assembly, intercellular connectivity is likely to be greater between cells within the same layer than between the cells of adjacent layers. Techniques for enhancing the formation of physical and electromechanical connections between layers include the use of graphene oxide (GO)-based thin films, which improved not only adhesion but also electrical coupling, maturation and cell organization (165), as well as the Tissue-Velcro platform, in which cardiac cells are cultured on 2D cell meshes that incorporate a microfabricated hook-and-loop system. After layering, the hooks and loops of adjacent layers interlocked and the hCMP contracted in response to electrical stimulation (166). The vascularization of multilayered hCMPs can be promoted by sandwiching vascular cells between the CM layers (167, 168). Coherent vascular networks have been generated in stacked hCMPs by using resected tissue as a vascular bed. When the resected vasculature was overlaid with sheets of cardiac cells (including ECs), connected to a bioreactor, and perfused with culture medium, ECs in the cardiac-cell layers connected to capillaries in the vascular bed and formed tubular structures. The vascularized hCMPs could survive after transplantation into the necks of rats via blood vessel anastomosis (169).

#### *In vitro* Perfusion

Techniques for maintaining adequate oxygen and nutrient availability during the manufacturing process include the development of cartridges that reproduce the convective-diffusive properties of oxygen transportation present in native myocardial tissue (170). Another approach is culturing cells on a simulated capillary network with medium containing an oxygen carrier (perfluorocarbon) that mimics hemoglobin (171, 172). The hemoglobin mimic significantly increased the expression of cardiac markers and improved the contractile performance of constructs composed of primary neonatal CMs and fibroblasts. Small intestinal submucosa could be utilized to enhance perfusion with rat primary endothelial cells forming a network in pre-existing vessel structures (173). AngioChip technology (174) incorporates a perfusable, 3D microchannel network that recapitulates the native vascular interface and, because it is compatible with current practices in both laboratories and industry, can be adapted to produce a variety of tissue types. Perfusion can also be improved by culturing cardiac tissue constructs under dynamic conditions [e.g., on a rocking platform; (158)]. This approach was used during the manufacture of large (2 cm × 4 cm) patches for subsequent testing in a swine MI model and was associated with improvements in not only cell viability, but also in measures of hCMP maturation (23). Notably, some evidence suggests that the contractile properties of hCMPs can be enhanced by perfusing them in rhythmic pulses, rather than a continuous static flow (175).

#### Engineered Vascular Networks

Rather than relying solely on infiltration from the native circulatory system, thicker hCMPs will likely require at least some amount of engineered vascularity before transplantation. Vascularization can be induced during the manufacturing process by encapsulating a sacrificial gelatin mesh in scaffold material and then melting the gelatin mesh away, leaving behind a network of interconnected microfluidic channels. When seeded with human microvascular ECs, the sacrificial scaffolds produced a rudimentary endothelial network (176). An alternative strategy mimics the endogenous angiogenic process by using a sustained-release preparation of the angiogenic factor thymosin β to promote and guide the outgrowth of vessels from explanted arteries and veins to form a capillary bed within a hydrogel scaffold (177). Vessel growth can also be directed with micropatterned polyglycerol sebacate scaffolds. After transplantation, host blood cells infiltrated into the microvessels as the scaffold degraded (178). Micropatterning has also been used to organize ECs into “cords,” which guide the formation of capillaries that integrate with the host tissue after transplantation (179).

One of the novel reports of 3D printed vessels used a thermal inkjet printer to print mixtures of human microvascular ECs and fibrin, forming micro-sized fibrin channels lined with confluent cells (180). Vascular networks have also been bioprinted with an advanced extrusion system that produced a sheath of photoactive, cell-laden bioink around an alginate core. After UV crosslinking, the alginate was dissolved with a Ca^2+^-chelating agent, allowing the cells to proliferate, spread, and form a perfusable biomimetic vascular network (140). However, the penetration of UV radiation is limited. Polymerization can be induced at greater depths via enzymatic reactions, such as the thrombin-induced cleavage of fibrinogen into fibrin. This strategy has been combined with the co-printing of vascular and cellular inks in cast ECM material to generate markedly thick (>1 cm) engineered osteogenic tissues (181).

### Transplantation of hCMP Constructs

#### hCMP Delivery Methods

Delivery methods for cells and tissues could be categorized into invasive and non-invasive approaches, both often featuring low engraftment rates (12). Transplantation of hCMPs mainly falls into invasive delivery, requiring open-chest surgery, and suturing/attaching the patch onto the epicardium (23, 62). This method typically offers an enhanced engraftment and survival rate compared with cell injection into the injured myocardium. The widespread application of intra-myocardial cell injections has demonstrated a poor cell retention rate with induced damages to both exogenous and host cells due to a dramatic increase in flow velocity and shear stress (182). Studies have shown that repeated cell injections increase the therapeutic effects (183). However, invasive delivery methods are not optimal for repeated applications due to requirements of dedicated facilities and highly trained staff to perform open-chest surgery. As a result, invasive delivery of cells and hCMPs has challenged clinical translation of these therapies (184).

Alternatively, non-invasive delivery methods, such as intravascular (or intravenous) delivery and injectable hydrogel approaches, have been tested. Intravascular delivery has shown recirculation and redistribution of injected cells to other organs besides the target site (6). These methods have demonstrated improved cardiac function potentially due to the paracrine signaling mechanism as previously discussed (185). This method has the advantages of being non-invasive and possibility of repeated administrations, qualifying them a good choice from a clinical viewpoint (184). Recently, a novel minimally invasive method demonstrated epicardial delivery of hydrogels through the pericardial space (186). A pericardial device (catheter) delivers the hydrogel components through separate (coaxial) lumens and combines them after exiting the device, forming a stable hydrogel construct between the pericardium and epicardium layers. This method could help minimize the risks of extensive myocardial injury, thrombotic occlusion, and arrhythmia. Injectable hydrogels have been also increasingly tested as a minimally (or non) invasive delivery approach for cardiac patch systems (187, 188). In a clinical study, acellular alginate-based hydrogels were injected in MI patients, demonstrating the preservation of the LV indices and ejection fraction (189). However, a more recent large clinical trial, investigating the effect of injectable alginate hydrogels in patients with advanced HF, reported about 9% death within 30 days post-injection, while the control group had no fatalities (190). Therefore, while significant progress has been made in developing injectable cardiac patch systems, more efforts are required for their further improvement for efficient clinical use. These include further enhancement in biomechanical and biochemical properties of the hydrogel constructs to mimic the native tissue, improving cell viability and biomolecules activity, controlled degradation and immune response, and enhancement in the *in vivo* tracking of the patch (187, 191, 192).

#### Animal Models to Test hCMPs

Preclinical studies have utilized different animal models, including mice, rats, guinea pigs, swine, and non-human primates (Table 1). Choice of a suitable animal model with high predictive validity for safety profile and therapeutic outcomes is crucial for the clinical translational purposes. Initial studies of therapeutic efficacy of transplanted human cells or cardiac tissues were performed in immunocompromised rodents, including athymic rats and severe combined immunodeficiency mice, due to genetic manipulations and easy handling. However, the disparity of anatomy and physiology between rodents and human dampens the reliability of therapeutic outcomes (197). Subsequent preclinical studies utilize large animal models, including non-human primates and swine, demonstrating remuscularization and therapeutic efficacy with safety concerns of ventricular arrhythmias (42, 196, 198). Large animal models are more reliable and relevant for preclinical studies compared with rodent models. However, the high cost associated with large animal studies would limit their applications (41).

**Table 1:** Summary of various hCMP studies conducted in animal models of heart injuries.

**Animal model**	**Cell type and number**	**Delivery method/Material**	**Vascularization**	**Observations**	**Follow up**
NOD/SCID gamma mice with MI (101)	Five Spheroids composed of hiPSC-CMs (2 × 10^5^/spheroid)	Engineered fibrin cardiac patch	N/A	Improved LVEF and FS; enhanced engraftment	4 weeks
NOD/SCID gamma mice with MI (163)	1 × 10^6^ hiPSC-CM	Engineered fibrin cardiac patch containing nanoparticles releasing CHIR99021 and FGF1	N/A	Enhanced cardiac function; reduced infarct size; increased engraftment with enhanced cell cycle activity of hiPSC-CMs	4 weeks
Guinea pigs with cryoinjury (193)	1 × 10^8^ hESC-CMs	IM	N/A	Improved mechanical function; reduced incidence of arrhythmias	4 weeks
Guinea pigs with MI (52)	5 × 10^6^ hiPSC-CM and 2 × 10^6^ hiPSC-ECs	Engineered fibrin cardiac patch	Inclusion of iPSC-ECs	Improve left ventricular function by 31%; remuscularization; vascularization; electrical coupling with host myocardium	28 days
Rats with MI (35)	4 × 10^7^ Fetal rat ventricular muscle	Engineered gelatin cardiac patch	N/A	No significant change in cardiac function; surviving grafts enhanced angiogenesis	5 weeks
Rats with MI (36)	3 × 10^5^ Fatal rat CMs	Engineered alginate cardiac patch	N/A	No significant change; Engraftment with intensive neovascularization; integration; attenuate LV dilatation	9 weeks
Normal athymic rats Sprague Dawley (56)	ESC-CMs and iPSC-CMs; HUVEC; MSCs; MEFs	Engineered collagen cardiac patch	Inclusion of stromal supporting cells	Form grafts containing microvessels; Enhanced formation of vessel-like structures	1 week
Rats with MI (88)	1 × 10^6^ Bone marrow mesenchymal stem cells	Engineered silk fibroin/hyaluronic acid cardiac patch	N/A	Therapeutic efficacy; improved LV wall thickness; high viability; neovascularization	8 weeks
Nude athymic rats with MI (39, 194)	2.5 × 10^6^ hESC-CMs	Engineered collagen cardiac patch	N/A	No significant changes of LVEF; Preserved heart function revealed by tagged magnetic resonance imaging; high engraftment	4 weeks
Athymic rats with MI (40)	2.2 × 10^6^ iPSC-CMs; 3.4 × 10^5^ human pericytes	Engineered fibrin cardiac patch	Inclusion of human pericytes	Improved cardiac function; reduced infarct size; engraft onto host heart	4 weeks
Rats with acute MI (139)	Acellular	Decellularized porcine myocardial slice	Utilization of decellularized porcine myocardium mimicking native ECM	Improved cardiac function; attach to host myocardium; prevent LV wall thinning; vascularization	4 weeks
Rats and porcine with MI (152)	Acellular; synthetic cardiac stromal cells	Cardiac patch composed of a decellularized porcine myocardium		Enhanced cardiac recovery with reduced scar and promoted angiogenesis in rat model; therapeutic efficacy in porcine model	3 weeks
Porcine with MI (113)	1.5 × 10^7^ Skeletal myoblasts from mini-pigs	Cell sheets covered with omentum flap	Utilization of omentum flap for revasularization	Improved cardiac function; Reduced infarct size; increased angiogenesis	8 weeks
Porcine minipigs with MI (114)	iPSC-CMs	Cell sheets	N/A	Improved cardiac performance and attenuated left ventricular remodeling; poor engraftment	8 weeks
Normal porcine mini-pigs (115)	iPSC-CMs	Cell sheets with an omentum flap	Inclusion of an omentum flap	Enhanced survival and engraftment; rich vasculature	8 weeks
Immunosuppressed Yorkshire pigs with MI (51)	4 × 10^6^ hiPSC-VC (ECs and SMCs)	Engineered fibrin cardiac patch	Inclusion of iPSC-VCs (ECs and SMCs)	Increased BZ contractile function and ATP turnover rate with attenuated regional wall stress, neovascularization and improved BZ perfusion	4 weeks
Porcine with acute MI (50)	6 × 10^6^ total iPSC-cardiovascular cells (CMs, ECs, and SMCs; each 2 × 10^6^)	IM + fibrin patch containing IGF	Inclusion of iPSC-ECs, SMCs	Improved left ventricular function; reduced infarct size; Integration with host myocardium; vascularization;	4 weeks
Porcine with ICM (116)	5 × 10^6^ iPSC-CMs	Cell sheets with omental flap	Utilization of omentum flap	Enhanced therapeutic effects and survival;	3 months
Porcine with MI (23)	iPSC-Cardiac cells (4 × 10^6^ CMs, 2 × 10^6^ ECs, and 2 × 10^6^ SMCs)	Engineered fibrin cardiac patch	Inclusion of iPSC-ECs, -SMCs	Improved left ventricular function; reduced infarct size; reduced LV wall stress; no significant changes in arrhythmogenicity	4 weeks
Porcine with MI (195)	Acellular; exosomes derived from iPSC-Cardiac cells (iPSC-CMs, -ECs, -SMCs)	IM	N/A	Improve myocardial recovery without increasing the frequency of arrhythmogenic	4 weeks
Non-human primate *Macaca nemestrina* with MI (41)	1 × 10^9^ (1 billion) hESC-CMs	IM	N/A	Remuscularization; vascularization; electromechanical coupling; arrhythmias	3 months
Non-human primate *Macaca fascicularis* with MI (196)	1 × 10^9^ iPSC-CMs	IM	N/A	Improved cardiac function; Survive for 12 weeks; electrical coupling; ventricular tachycardia (transient)	12 weeks
Non-human primate macaque monkeys with MI (42)	~750 million hESC-CMs	IM	N/A	Improved cardiac function; formation of electromechanical junctions; ventricular arrhythmias	3 months
One patient with severe heart failure (90)	4 × 10^6^ hESC-derived Isl-1^+^ SSEA-1^+^ cells	Engineered fibrin cardiac patch	N/A	Symptomatically improved LVEF; new-onset contractility; no complications	3 months
Seven patients with ischemic cardiomyopathy (UMIN000008013) (118)	3 × 10^8^ autologous skeletal muscle	Cell sheet	N/A	Improved LVEF; improved patient status	26 weeks
Six patients with severe ischemic left ventricular dysfunction (NCT02057900) (91)	8.2 × 10^6^ hESC-derived cardiovascular progenitors	Engineered fibrin cardiac patch	N/A	Uneventful recoveries; no safety concerns, such as tumor, arrhythmias, alloimmunization	1 year
Four patients with dilated cardiomyopathy (UMIN000000660) (119)	4.5–7.5 × 10^8^ autologous skeletal muscle	Cell sheet	N/A	Improved LVEF in two patients; reduced cardiac hypertrophy	3 months

*FGF1, fibroblast growth factor 1; LVEF, left ventricular ejection fraction; FS, fractional shortening; IM, intra-myocardial delivery; HUVECs, human umbilical vein endothelial cells; MSCs, human marrow stromal cells; MEFs, mouse embryonic fibroblasts; IGF, insulin growth factor; ICM, ischemic cardiomyopathy*.

#### Mechanisms of Action of hCMPs

The ultimate goal of cardiac patch transplantation is to replace the injured myocardium with exogenously functional cardiac muscle. Preclinical studies from mice (199, 200), rats (201), guinea pigs (52, 193), porcine (50), and non-human primates (41) have demonstrated some degree of myocardial remuscularization of the fibrotic scar tissue with transplanted cardiac tissues. The clinical translation of this approach has met some challenges, first of which is the requirement of large quantities of exogenous CMs (and/or other cardiac cells) used to replenish lost tissue. Robust protocols for CM differentiation and scalability of CM production, together with cryopreservation and retrieval procedures following current good manufacturing practice (cGMP), have enabled generation of well-characterized PSC-CMs as an off-the-shelf therapy (202). Furthermore, it remains a critical issue to ensure long-term graft retention for maximal therapeutic efficacy based on the hypothesis that contractile force is correlated to the electromechanically integrated CMs (202). Gene manipulation has been adopted to increase cell retention. Overexpression of CCND2 (cyclin D2), a cell cycle activator, increased cell cycle activity and proliferation rate in hiPSC-CMs, thus improving engraftment rate from average 10 to 25% with a significant remuscularization of injured myocardium in mice (203). CM retention could also be enhanced via co-administration of pro-survival factors, such as Matrigel, cyclosporine A, pinacidil, ZVAD-fmk, insulin-like growth factor-1 (IGF-1), CHIR99021, and fibroblast growth factor 1(FGF1) (50, 82, 163).

The lack of electromechanical coupling between hCMPs and host tissue is another challenge on the way of clinical translation of patch-based therapies. Super-aligned carbon nanotubes were utilized during the fabrication of cardiac tissues to enhance electrophysiological homogeneity due to the anisotropic conductivity of their aligned structure (204). In another study, electrospun nanofibrous scaffolds with enhanced conductivity were demonstrated to promote electrical coupling of the patch, showing potential for fabrication of clinically relevant hCMP products (205). One major aspect that must be carefully studied, when working on electromechanical integration of exogenous CMs with host myocardium is the likely potential to trigger arrhythmias, as discussed in the previous sections (41).

Low CM survival rates, as little as 10%, have been usually associated with functional benefits from hCMP transplantations (39, 51, 194). Patch constructs composed of non-CMs have also shown improved cardiac function (51). These data collectively suggest an alternative mechanism of action, based on paracrine signaling, where cell secreted signals, including extracellular vesicles, mediate cardiac repair via increasing CM survival and neovascularization and decreasing apoptosis and inflammation (206). Paracrine signaling was further evidenced by a study where exosomes secreted from combination of iPSC-derived CMs, ECs, and SMCs yielded cardioprotective effects similar to that obtained from direct cells injection in a porcine model of MI (195). Further, acellular hCMPs composed of non-viable/irradiated cells (39) or decellularized porcine myocardial tissues (139) have shown therapeutic benefits after transplantation, through the proposed mechanism of mechanical stabilization. Thus, the therapeutic benefits associated with hCMP transplantation could be a combination of any or all of mechanisms including remuscularization, paracrine signaling, and mechanical stabilization (202, 207).

#### Safety Concerns

Occurrence of arrhythmias is a critical safety concern for clinical applicability of cardiac patch therapies. Sine large animal models more closely approximate the human heart's physiology, function, and anatomy, they have been increasingly used to assess arrhythmias in cardiac patch studies (41). Preclinical studies of non-human primates demonstrated transient non-fatal arrhythmias during 2 weeks after transplantation, followed by a subsequent decrease in those irregularities (41, 196). Ventricular arrhythmias occurred in porcine models have been more frequent and lethal than those in non-human primate models (2 out of every 7 pigs) (198). It is currently viewed that disorder of impulse generation at the interface between engrafted CMs and host myocardium could induce ventricular arrhythmias (42, 198). Further investigation of arrhythmic complications will be necessary before translation into patients.

Autologous cell transplantation offers an advantageous option based on the hypothesis that autologous cell transplantation could induce no immunological response due to the acceptable human leukocyte antigens (HLA) match. However, accumulating evidence prove that the clinical translation of autologous cells could become complicated by limitations, such as variability from batch to batch, requiring a detailed characterization and quality control of each batch. The initial hypothesis that transplantation of autologous iPSC derivatives would induce no immunogenicity has been challenged by some recent studies (70, 71).

For a long time in clinical practice, inhibition of the immunological responses has been mainly through the use of immunosuppressive drugs, such as cyclosporine, dexamethasone, and FK-506. With side effects induced by such immunosuppressants, there is a trade-off between efficacy and toxicity. High-dose immunosuppression would result in toxicity and associated complications, while low-dose immunosuppression would lead to allograft rejection. The choice of transient or chronic exposure of immunosuppressants would be based on the presumed mechanism of action, as discussed previously (184, 208). In addition, immunological tolerance could be induced via host conditioning by activation/adoptive transfer of regulatory T cells (209). Short-term administration of nanobiologics targeting macrophages could benefit long-term allograft survival (210). Other strategies have been adopted to reduce immunogenicity. Modern genome-editing tools [e.g., zinc-finger nucleases (ZFNs), transcription activator-like effector nucleases (TALENs), or the clustered regulatory interspaced short palindromic repeat (CRISPR)/Cas-9 system; (37, 211, 212)] have enabled the development of minimally immunogenic iPSC lines by knocking out key components of major histocompatibility complexes (MHCs) I and II [Beta2 microglobulin (B2M) and MHC II transactivator (CIITA)] (213). This is done by combining MHC gene inactivation with the overexpression of CD47, a ubiquitous membrane protein that directly regulates T-cell immunity (214), and by disrupting the genes for HLA (215). These genetically modified hiPSCs could be used to generate a stockpile of “off-the-shelf” cardiac cells and hCMPs for administration to patients in emergency situations without the need for concomitant immunosuppressive therapies.

##### Summary—Current Challenges and Future Perspectives

The potential benefits of hCMPs for the treatment of myocardial injury and disease are readily observable in preclinical studies, and at least one small study in patients is currently underway (120). Conventional methods for manufacturing hCMPs include suspending cells in scaffolds of biocompatible material or growing 2D sheets in culture and stacking them to form multilayered constructs. More advanced technologies, such as micropatterning and CAD-guided 3D printing with bioinks have given researchers the tools to control the architecture of hCMPs at resolutions that match the scale of interactions between individual cells or between cells and the ECM. Most studies, however, have been conducted with hCMPs at relatively small scales that are not suitable for administration to patients. The size and (especially) thickness of hCMPs are often restricted by the diffusion limitations of oxygen, nutrients, and other biologically active molecules, which requires CMs to be within 100–200 μm distance from the capillaries. Thus, the clinical translation of hCMP technology will require the development of protocols for manufacturing larger and thicker constructs that are adequately vascularized and fully couple with the circulatory and electromechanical systems of the native myocardium.

## Author Contributions

LW, VS, and JZ contributed to the overall design of the manuscript, collecting the data, and writing the article. JZ supervised the entire manuscript preparation process, figures design and creation, obtaining the necessary permits, etc. All authors contributed to the article and approved the submitted version.

## Conflict of Interest

The authors declare that the research was conducted in the absence of any commercial or financial relationships that could be construed as a potential conflict of interest.

## References

[1] Laslett LJ Alagona P Jr. Clark BA III Drozda JP Saldivar F Wilson SR. The worldwide environment of cardiovascular disease: prevalence, diagnosis, therapy, and policy issues: a report from the American College of Cardiology. J Am Coll Cardiol. (2012) 60(Suppl. 25):S1–49. 10.1016/j.jacc.2012.11.002 23257320

[2] Tzatzalos E Abilez OJ Shukla P Wu JC. Engineered heart tissues and induced pluripotent stem cells: macro- and microstructures for disease modeling, drug screening, and translational studies. Adv Drug Deliv Rev. (2016) 96:234–44. 10.1016/j.addr.2015.09.010 26428619 PMC4698222

[3] Evans MA Sano S Walsh K. Cardiovascular disease, aging, and clonal hematopoiesis. Annu Rev Pathol. (2020) 15:419–38. 10.1146/annurev-pathmechdis-012419-032544 31689371 PMC7104598

[4] Bergmann O Zdunek S Felker A Salehpour M Alkass K Bernard S. Dynamics of cell generation and turnover in the human heart. Cell. (2015) 161:1566–75. 10.1016/j.cell.2015.05.026 26073943

[5] Travers JG Kamal FA Robbins J Yutzey KE Blaxall BC. Cardiac fibrosis: the fibroblast awakens. Circ Res. (2016) 118:1021–40. 10.1161/CIRCRESAHA.115.306565 26987915 PMC4800485

[6] Zhang J Zhu W Radisic M Vunjak-Novakovic G. Can we engineer a human cardiac patch for therapy? Circ Res. (2018) 123:244–65. 10.1161/CIRCRESAHA.118.311213 29976691 PMC7250155

[7] Parsa H Ronaldson K Vunjak-Novakovic G. Bioengineering methods for myocardial regeneration. Adv Drug Deliv Rev. (2016) 96:195–202. 10.1016/j.addr.2015.06.012 26150344 PMC4698189

[8] Fujita B Zimmermann WH. Myocardial tissue engineering for regenerative applications. Curr Cardiol Rep. (2017) 19:78. 10.1007/s11886-017-0892-4 28752277

[9] Pomeroy JE Helfer A Bursac N. Biomaterializing the promise of cardiac tissue engineering. Biotechnol Adv. (2020) 42:107353. 10.1016/j.biotechadv.2019.02.009 30794878 PMC6702110

[10] Orlic D Kajstura J Chimenti S Jakoniuk I Anderson SM Li B. Bone marrow cells regenerate infarcted myocardium. Nature. (2001) 410:701–5. 10.1038/35070587 11287958

[11] Nygren JM Jovinge S Breitbach M Säwén P Röll W Hescheler J. Bone marrow-derived hematopoietic cells generate cardiomyocytes at a low frequency through cell fusion, but not transdifferentiation. Nat Med. (2004) 10:494–501. 10.1038/nm1040 15107841

[12] Nguyen PK Rhee JW Wu JC. Adult stem cell therapy and heart failure, 2000 to 2016: a systematic review. JAMA Cardiol. (2016) 1:831–41. 10.1001/jamacardio.2016.2225 27557438 PMC5349705

[13] Ogle BM Bursac N Domian I Huang NF Menasché P Murry CE. Distilling complexity to advance cardiac tissue engineering. Sci Transl Med. (2016) 8:342ps13. 10.1126/scitranslmed.aad2304 27280684 PMC4959426

[14] Goldfracht I Efraim Y Shinnawi R Kovalev E Huber I Gepstein A. Engineered heart tissue models from hiPSC-derived cardiomyocytes and cardiac ECM for disease modeling and drug testing applications. Acta Biomater. (2019) 92:145–59. 10.1016/j.actbio.2019.05.016 31075518

[15] Meyer T Tiburcy M Zimmermann WH. Cardiac macrotissues-on-a-plate models for phenotypic drug screens. Adv Drug Deliv Rev. (2019) 140:93–100. 10.1016/j.addr.2019.03.002 30902615

[16] Turnbull IC Mayourian J Murphy JF Stillitano F Ceholski DK Costa KD. Cardiac tissue engineering models of inherited and acquired cardiomyopathies. Methods Mol Biol. (2018) 1816:145–59. 10.1007/978-1-4939-8597-5_11 29987817 PMC6561092

[17] Zhao Y Rafatian N Feric NT Cox BJ Aschar-Sobbi R Wang EY. A platform for generation of chamber-specific cardiac tissues and disease modeling. Cell. (2019) 176:913–27.e18. 10.1016/j.cell.2018.11.042 30686581 PMC6456036

[18] Giacomelli E Meraviglia V Campostrini G Cochrane A Cao X van Helden RWJ. Human-iPSC-Derived cardiac stromal cells enhance maturation in 3D cardiac microtissues and reveal non-cardiomyocyte contributions to heart disease. Cell Stem Cell. (2020) 26:862–79.e11. 10.1016/j.stem.2020.05.004 32459996 PMC7284308

[19] Shadrin IY Allen BW Qian Y Jackman CP Carlson AL Juhas ME. Cardiopatch platform enables maturation and scale-up of human pluripotent stem cell-derived engineered heart tissues. Nat Commun. (2017) 8:1825. 10.1038/s41467-017-01946-x 29184059 PMC5705709

[20] Weinberger F Mannhardt I Eschenhagen T. Engineering cardiac muscle tissue: a maturating field of research. Circ Res. (2017) 120:1487–500. 10.1161/CIRCRESAHA.117.310738 28450366

[21] Eschenhagen T Fink C Remmers U Scholz H Wattchow J Weil J. Three-dimensional reconstitution of embryonic cardiomyocytes in a collagen matrix: a new heart muscle model system. Faseb J. (1997) 11:683–94. 10.1096/fasebj.11.8.9240969 9240969

[22] Lian X Hsiao C Wilson G Zhu K Hazeltine LB Azarin SM. Robust cardiomyocyte differentiation from human pluripotent stem cells via temporal modulation of canonical Wnt signaling. Proc Natl Acad Sci USA. (2012) 109:E1848–57. 10.1073/pnas.1200250109 22645348 PMC3390875

[23] Gao L Gregorich ZR Zhu W Mattapally S Oduk Y Lou X. Large cardiac muscle patches engineered from human induced-pluripotent stem cell–derived cardiac cells improve recovery from myocardial infarction in swine. Circulation. (2018) 137:1712–30. 10.1161/CIRCULATIONAHA.117.030785 29233823 PMC5903991

[24] Shimizu I Minamino T. Physiological and pathological cardiac hypertrophy. J Mol Cell Cardiol. (2016) 97:245–62. 10.1016/j.yjmcc.2016.06.001 27262674

[25] Cimetta E Godier-Furnémont A Vunjak-Novakovic G. Bioengineering heart tissue for *in vitro* testing. Curr Opin Biotechnol. (2013) 24:926–32. 10.1016/j.copbio.2013.07.002 23932513 PMC3783612

[26] Zhou P Pu WT. Recounting cardiac cellular composition. Circ Res. (2016) 118:368–70. 10.1161/CIRCRESAHA.116.308139 26846633 PMC4755297

[27] Shimizu T Yamato M Kikuchi A Okano T. Cell sheet engineering for myocardial tissue reconstruction. Biomaterials. (2003) 24:2309–16. 10.1016/S0142-9612(03)00110-8 12699668

[28] Bursac N Loo Y Leong K Tung L. Novel anisotropic engineered cardiac tissues: studies of electrical propagation. Biochem Biophys Res Commun. (2007) 361:847–53. 10.1016/j.bbrc.2007.07.138 17689494 PMC2570036

[29] Banerjee I Fuseler JW Price RL Borg TK Baudino TA. Determination of cell types and numbers during cardiac development in the neonatal and adult rat and mouse. Am J Physiol Heart Circ Physiol. (2007) 293:H1883–91. 10.1152/ajpheart.00514.2007 17604329

[30] Fleischer S Vunjak-Novakovic G. Cardiac tissue engineering: from repairing to modeling the human heart. In: Reis RL editor. Encyclopedia of Tissue Engineering and Regenerative Medicine. Oxford: Academic Press (2019). p. 131–44. 10.1016/B978-0-12-801238-3.65543-5

[31] Wobma H Vunjak-Novakovic G. Tissue engineering and regenerative medicine 2015: a year in review. Tissue Eng Part B Rev. (2016) 22:101–13. 10.1089/ten.teb.2015.0535 26714410 PMC4817587

[32] Soonpaa MH Koh GY Klug MG Field LJ. Formation of nascent intercalated disks between grafted fetal cardiomyocytes and host myocardium. Science. (1994) 264:98–101. 10.1126/science.8140423 8140423

[33] Souren JE Schneijdenberg C Verkleij AJ Van Wijk R. Factors controlling the rhythmic contraction of collagen gels by neonatal heart cells. In Vitro Cell Dev Biol. (1992) 28a(3 Pt 1):199–204. 10.1007/BF02631092 1582995

[34] Souren JE Peters RC Van Wijk R. Collagen gels populated with rat neonatal heart cells can be used for optical recording of rhythmic contractions which also show ECG-like potentials. Experientia. (1994) 50:712–6. 10.1007/BF01919368 8070530

[35] Li RK Jia ZQ Weisel RD Mickle DA Choi A Yau TM. Survival and function of bioengineered cardiac grafts. Circulation. (1999) 100(Suppl. 19):Ii63–9. 10.1161/01.CIR.100.suppl_2.II-63 10567280

[36] Leor J Aboulafia-Etzion S Dar A Shapiro L Barbash IM Battler A. Bioengineered cardiac grafts: a new approach to repair the infarcted myocardium? Circulation. (2000) 102(19 Suppl. 3):Iii56–61. 10.1161/01.CIR.102.suppl_3.III-56 11082363

[37] Chen IY Matsa E Wu JC. Induced pluripotent stem cells: at the heart of cardiovascular precision medicine. Nat Rev Cardiol. (2016) 13:333–49. 10.1038/nrcardio.2016.36 27009425 PMC5917945

[38] Caspi O Lesman A Basevitch Y Gepstein A Arbel G Habib IH. Tissue engineering of vascularized cardiac muscle from human embryonic stem cells. Circ Res. (2007) 100:263–72. 10.1161/01.RES.0000257776.05673.ff 17218605

[39] Riegler J Tiburcy M Ebert A Tzatzalos E Raaz U Abilez OJ. Human engineered heart muscles engraft and survive long term in a rodent myocardial infarction model. Circ Res. (2015) 117:720–30. 10.1161/CIRCRESAHA.115.306985 26291556 PMC4679370

[40] Wendel JS Ye L Tao R Zhang J Zhang J Kamp TJ. Functional effects of a tissue-engineered cardiac patch from human induced pluripotent stem cell-derived cardiomyocytes in a rat infarct model. Stem Cells Transl Med. (2015) 4:1324–32. 10.5966/sctm.2015-0044 26371342 PMC4622407

[41] Chong JJ Yang X Don CW Minami E Liu YW Weyers JJ. Human embryonic-stem-cell-derived cardiomyocytes regenerate non-human primate hearts. Nature. (2014) 510:273–7. 10.1038/nature13233 24776797 PMC4154594

[42] Liu YW Chen B Yang X Fugate JA Kalucki FA Futakuchi-Tsuchida A. Human embryonic stem cell-derived cardiomyocytes restore function in infarcted hearts of non-human primates. Nat Biotechnol. (2018) 36:597–605. 10.1038/nbt.4162 29969440 PMC6329375

[43] Nunes SS Miklas JW Liu J Aschar-Sobbi R Xiao Y Zhang B. Biowire: a platform for maturation of human pluripotent stem cell-derived cardiomyocytes. Nat Methods. (2013) 10:781–7. 10.1038/nmeth.2524 23793239 PMC4071061

[44] Zhang D Shadrin IY Lam J Xian HQ Snodgrass HR Bursac N. Tissue-engineered cardiac patch for advanced functional maturation of human ESC-derived cardiomyocytes. Biomaterials. (2013) 34:5813–20. 10.1016/j.biomaterials.2013.04.026 23642535 PMC3660435

[45] Yang X Pabon L Murry CE. Engineering adolescence: maturation of human pluripotent stem cell-derived cardiomyocytes. Circ Res. (2014) 114:511–23. 10.1161/CIRCRESAHA.114.300558 24481842 PMC3955370

[46] Ruan JL Tulloch NL Razumova MV Saiget M Muskheli V Pabon L. Mechanical stress conditioning and electrical stimulation promote contractility and force maturation of induced pluripotent stem cell-derived human cardiac tissue. Circulation. (2016) 134:1557–67. 10.1161/CIRCULATIONAHA.114.014998 27737958 PMC5123912

[47] Guo XM Zhao YS Chang HX Wang CY E LL Zhang XA. Creation of engineered cardiac tissue in *vitro* from mouse embryonic stem cells. Circulation. (2006) 113:2229–37. 10.1161/CIRCULATIONAHA.105.583039 16651472

[48] Talman V Kivelä R. Cardiomyocyte-endothelial cell interactions in cardiac remodeling and regeneration. Front Cardiovasc Med. (2018) 5:101. 10.3389/fcvm.2018.00101 30175102 PMC6108380

[49] Zakharova IS Zhiven MK Saaya SB Shevchenko AI Smirnova AM Strunov A. Endothelial and smooth muscle cells derived from human cardiac explants demonstrate angiogenic potential and suitable for design of cell-containing vascular grafts. J Transl Med. (2017) 15:54. 10.1186/s12967-017-1156-1 28257636 PMC5336693

[50] Ye L Y.-Chang H Xiong Q Zhang P Zhang L Somasundaram P. Cardiac repair in a porcine model of acute myocardial infarction with human induced pluripotent stem cell-derived cardiovascular cells. Cell Stem Cell. (2014) 15:750–61. 10.1016/j.stem.2014.11.009 25479750 PMC4275050

[51] Xiong Q Ye L Zhang P Lepley M Tian J Li J. Functional consequences of human induced pluripotent stem cell therapy: myocardial ATP turnover rate in the *in vivo* swine heart with postinfarction remodeling. Circulation. (2013) 127:997–1008. 10.1161/CIRCULATIONAHA.112.000641 23371930 PMC3980462

[52] Weinberger F Breckwoldt K Pecha S Kelly A Geertz B Starbatty J. Cardiac repair in guinea pigs with human engineered heart tissue from induced pluripotent stem cells. Sci Transl Med. (2016) 8:363ra148. 10.1126/scitranslmed.aaf8781 27807283

[53] Choi KD Yu J Smuga-Otto K Salvagiotto G Rehrauer W Vodyanik M. Hematopoietic and endothelial differentiation of human induced pluripotent stem cells. Stem Cells. (2009) 27:559–67. 10.1002/stem.20080922 19259936 PMC2931800

[54] Shu B Xie JL Xu YB Yu JX Shi Y Liu J. Directed differentiation of skin-derived precursors into fibroblast-like cells. Int J Clin Exp Pathol. (2014) 7:1478–86. 24817943 PMC4014227

[55] Gao L Yang L Wang L Geng Z Wei Y Gourley G. Relationship between the efficacy of cardiac cell therapy and the inhibition of differentiation of human iPSC-derived nonmyocyte cardiac cells into myofibroblast-like cells. Circ Res. (2018) 123:1313–25. 10.1161/CIRCRESAHA.118.313094 30566050 PMC6309999

[56] Tulloch NL Muskheli V Razumova MV Korte FS Regnier M Hauch KD. Growth of engineered human myocardium with mechanical loading and vascular coculture. Circ Res. (2011) 109:47–59. 10.1161/CIRCRESAHA.110.237206 21597009 PMC3140796

[57] Zhang S Dutton JR Su L Zhang J Ye L. The influence of a spatiotemporal 3D environment on endothelial cell differentiation of human induced pluripotent stem cells. Biomaterials. (2014) 35:3786–93. 10.1016/j.biomaterials.2014.01.037 24485793 PMC3982910

[58] Patsch C Challet-Meylan L Thoma EC Urich E Heckel T O'Sullivan JF. Generation of vascular endothelial and smooth muscle cells from human pluripotent stem cells. Nat Cell Biol. (2015) 17:994–1003. 10.1038/ncb3205 26214132 PMC4566857

[59] Zhang J Chu LF Hou Z Schwartz MP Hacker T Vickerman V. Functional characterization of human pluripotent stem cell-derived arterial endothelial cells. Proc Natl Acad Sci USA. (2017) 114:E6072–8. 10.1073/pnas.1702295114 28696312 PMC5544294

[60] Zhang J Tao R Campbell KF Carvalho JL Ruiz EC Kim GC. Functional cardiac fibroblasts derived from human pluripotent stem cells via second heart field progenitors. Nat Commun. (2019) 10:2238. 10.1038/s41467-019-09831-5 31110246 PMC6527555

[61] Giacomelli E Bellin M Orlova VV Mummery CL. Co-differentiation of human pluripotent stem cells-derived cardiomyocytes and endothelial cells from cardiac mesoderm provides a three-dimensional model of cardiac microtissue. Curr Protoc Hum Genet. (2017) 95:21.9.1–21.9.22. 10.1002/cphg.46 29044469

[62] Gao L Kupfer ME Jung JP Yang L Zhang P Da Sie Y. Myocardial tissue engineering with cells derived from human-induced pluripotent stem cells and a native-like, high-resolution, 3-dimensionally printed scaffold. Circ Res. (2017) 120:1318–25. 10.1161/CIRCRESAHA.116.310277 28069694 PMC5392171

[63] Liau B Jackman CP Li Y Bursac N. Developmental stage-dependent effects of cardiac fibroblasts on function of stem cell-derived engineered cardiac tissues. Sci Rep. (2017) 7:42290. 10.1038/srep42290 28181589 PMC5299411

[64] Ronaldson-Bouchard K Ma SP Yeager K Chen T Song L Sirabella D. Advanced maturation of human cardiac tissue grown from pluripotent stem cells. Nature. (2018) 556:239–43. 10.1038/s41586-018-0016-3 29618819 PMC5895513

[65] Evans MJ Kaufman MH. Establishment in culture of pluripotential cells from mouse embryos. Nature. (1981) 292:154–6. 10.1038/292154a0 7242681

[66] Kim K Lerou P Yabuuchi A Lengerke C Ng K West J. Histocompatible embryonic stem cells by parthenogenesis. Science. (2007) 315:482–6. 10.1126/science.1133542 17170255

[67] Sayed N Liu C Wu JC. Translation of human-induced pluripotent stem cells: from clinical trial in a dish to precision medicine. J Am Coll Cardiol. (2016) 67:2161–76. 10.1016/j.jacc.2016.01.083 27151349 PMC5086255

[68] Yu J Vodyanik MA Smuga-Otto K Antosiewicz-Bourget J Frane JL Tian S. Induced pluripotent stem cell lines derived from human somatic cells. Science. (2007) 318:1917–20. 10.1126/science.1151526 18029452

[69] Huangfu D Osafune K Maehr R Guo W Eijkelenboom A Chen S. Induction of pluripotent stem cells from primary human fibroblasts with only Oct4 and Sox2. Nat Biotechnol. (2008) 26:1269–75. 10.1038/nbt.1502 18849973

[70] Zhao T Zhang ZN Rong Z Xu Y. Immunogenicity of induced pluripotent stem cells. Nature. (2011) 474:212–5. 10.1038/nature10135 21572395

[71] Kaneko S Yamanaka S. To be immunogenic, or not to be: that's the iPSC question. Cell Stem Cell. (2013) 12:385–86. 10.1016/j.stem.2013.03.008 23561437

[72] Kim EM Manzar G Zavazava N. Induced pluripotent stem cell-derived gamete-associated proteins incite rejection of induced pluripotent stem cells in syngeneic mice. Immunology. (2017) 151:191–7. 10.1111/imm.12722 28185259 PMC5706344

[73] Chen Z Zhao T Xu Y. The genomic stability of induced pluripotent stem cells. Protein Cell. (2012) 3:271–7. 10.1007/s13238-012-2922-8 22528751 PMC4875480

[74] Itskovitz-Eldor J Schuldiner M Karsenti D Eden A Yanuka O Amit M. Differentiation of human embryonic stem cells into embryoid bodies compromising the three embryonic germ layers. Mol Med. (2000) 6:88–95. 10.1007/BF03401776 10859025 PMC1949933

[75] Kehat I Kenyagin-Karsenti D Snir M Segev H Amit M Gepstein A. Human embryonic stem cells can differentiate into myocytes with structural and functional properties of cardiomyocytes. J Clin Invest. (2001) 108:407–14. 10.1172/JCI200112131 11489934 PMC209357

[76] He JQ Ma Y Lee Y Thomson JA Kamp TJ. Human embryonic stem cells develop into multiple types of cardiac myocytes: action potential characterization. Circ Res. (2003) 93:32–9. 10.1161/01.RES.0000080317.92718.99 12791707

[77] Osafune K Caron L Borowiak M Martinez RJ Fitz-Gerald CS Sato Y. Marked differences in differentiation propensity among human embryonic stem cell lines. Nat Biotechnol. (2008) 26:313–5. 10.1038/nbt1383 18278034

[78] Mummery CL Zhang J Ng ES Elliott DA Elefanty AG Kamp TJ. Differentiation of human embryonic stem cells and induced pluripotent stem cells to cardiomyocytes: a methods overview. Circ Res. (2012) 111:344–58. 10.1161/CIRCRESAHA.110.227512 22821908 PMC3578601

[79] Halloin C Schwanke K Löbel W Franke A Szepes M Biswanath S. Continuous WNT control enables advanced hPSC cardiac processing and prognostic surface marker identification in chemically defined suspension culture. Stem Cell Rep. (2019) 13:366–79. 10.1016/j.stemcr.2019.06.004 PMC6700605 31353227

[80] Devalla HD Schwach V Ford JW Milnes JT El-Haou S Jackson C. Atrial-like cardiomyocytes from human pluripotent stem cells are a robust preclinical model for assessing atrial-selective pharmacology. EMBO Mol Med. (2015) 7:394–410. 10.15252/emmm.201404757 25700171 PMC4403042

[81] Lee JH Protze SI Laksman Z Backx PH Keller GM. Human pluripotent stem cell-derived atrial and ventricular cardiomyocytes develop from distinct mesoderm populations. Cell Stem Cell. (2017) 21:179–94.e4. 10.1016/j.stem.2017.07.003 28777944

[82] Laflamme MA Chen KY Naumova AV Muskheli V Fugate JA Dupras SK. Cardiomyocytes derived from human embryonic stem cells in pro-survival factors enhance function of infarcted rat hearts. Nat Biotechnol. (2007) 25:1015–24. 10.1038/nbt1327 17721512

[83] Kattman SJ Witty AD Gagliardi M Dubois NC Niapour M Hotta A. Stage-specific optimization of activin/nodal and BMP signaling promotes cardiac differentiation of mouse and human pluripotent stem cell lines. Cell Stem Cell. (2011) 8:228–40. 10.1016/j.stem.2010.12.008 21295278

[84] Lian X Zhang J Azarin SM Zhu K Hazeltine LB Bao X. Directed cardiomyocyte differentiation from human pluripotent stem cells by modulating Wnt/β-catenin signaling under fully defined conditions. Nat Protoc. (2013) 8:162–75. 10.1038/nprot.2012.150 23257984 PMC3612968

[85] Kropp C Kempf H Halloin C Robles-Diaz D Franke A Scheper T. Impact of feeding strategies on the scalable expansion of human pluripotent stem cells in single-use stirred tank bioreactors. Stem Cells Transl Med. (2016) 5:1289–301. 10.5966/sctm.2015-0253 27369897 PMC5031176

[86] Ackermann M Kempf H Hetzel M Hesse C Hashtchin AR Brinkert K. Bioreactor-based mass production of human iPSC-derived macrophages enables immunotherapies against bacterial airway infections. Nat Commun. (2018) 9:5088. 10.1038/s41467-018-07570-7 30504915 PMC6269475

[87] Chen VC Ye J Shukla P Hua G Chen D Lin Z. Development of a scalable suspension culture for cardiac differentiation from human pluripotent stem cells. Stem Cell Res. (2015) 15:365–75. 10.1016/j.scr.2015.08.002 26318718 PMC4600677

[88] Chi NH Yang MC Chung TW Chen JY Chou NK Wang SS. Cardiac repair achieved by bone marrow mesenchymal stem cells/silk fibroin/hyaluronic acid patches in a rat of myocardial infarction model. Biomaterials. (2012) 33:5541–51. 10.1016/j.biomaterials.2012.04.030 22575829

[89] Menasché P Vanneaux V Fabreguettes JR Bel A Tosca L Garcia S. Towards a clinical use of human embryonic stem cell-derived cardiac progenitors: a translational experience. Eur Heart J. (2015) 36:743–50. 10.1093/eurheartj/ehu192 24835485

[90] Menasché P Vanneaux V Hagège A Bel A Cholley B Cacciapuoti I. Human embryonic stem cell-derived cardiac progenitors for severe heart failure treatment: first clinical case report. Eur Heart J. (2015) 36:2011–7. 10.1093/eurheartj/ehv189 25990469

[91] Menasché P Vanneaux V Hagège A Bel A Cholley B Parouchev A. Transplantation of human embryonic stem cell-derived cardiovascular progenitors for severe ischemic left ventricular dysfunction. J Am Coll Cardiol. (2018) 71:429–38. 10.1016/j.jacc.2017.11.047 29389360

[92] Qasim M Haq F Kang MH Kim JH. 3D printing approaches for cardiac tissue engineering and role of immune modulation in tissue regeneration. Int J Nanomedicine. (2019). 14:1311–33. 10.2147/IJN.S189587 30863063 PMC6388753

[93] Tomov ML Theus A Sarasani R Chen H Serpooshan V. 3D bioprinting of cardiovascular tissue constructs: cardiac bioinks. In: Serpooshan V Wu SM editors. Cardiovascular Regenerative Medicine: Tissue Engineering Clinical Applications. Cham: Springer International Publishing (2019). p. 63–77. 10.1007/978-3-030-20047-3_4

[94] Tomov ML Gil CJ Cetnar A Theus AS Lima BJ Nish JE. Engineering functional cardiac tissues for regenerative medicine applications. Curr Cardiol Rep. (2019) 21:105. 10.1007/s11886-019-1178-9 31367922 PMC7153535

[95] Nguyen DC Hookway TA Wu Q Jha R Preininger MK Chen X. Microscale generation of cardiospheres promotes robust enrichment of cardiomyocytes derived from human pluripotent stem cells. Stem Cell Rep. (2014) 3:260–8. 10.1016/j.stemcr.2014.06.002 25254340 PMC4175548

[96] Polonchuk L Chabria M Badi L Hoflack JC Figtree G Davies MJ. Cardiac spheroids as promising *in vitro* models to study the human heart microenvironment. Sci Rep. (2017) 7:7005. 10.1038/s41598-017-06385-8 28765558 PMC5539326

[97] Campbell M Chabria M Figtree GA Polonchuk L Gentile C. Stem cell-derived cardiac spheroids as 3D *in vitro* models of the human heart microenvironment. Methods Mol Biol. (2019) 2002:51–9. 10.1007/7651_2018_187 30159827

[98] Oltolina F Zamperone A Colangelo D Gregoletto L Reano S Pietronave S. Human cardiac progenitor spheroids exhibit enhanced engraftment potential. PLoS ONE. (2015) 10:e0137999. 10.1371/journal.pone.0137999 26375957 PMC4572703

[99] Zhao S Xu Z Wang H Reese BE Gushchina LV Jiang M. Bioengineering of injectable encapsulated aggregates of pluripotent stem cells for therapy of myocardial infarction. Nat Commun. (2016) 7:13306. 10.1038/ncomms13306 27786170 PMC5095349

[100] Bartulos O Zhuang ZW Huang Y Mikush N Suh C Bregasi A. ISL1 cardiovascular progenitor cells for cardiac repair after myocardial infarction. JCI Insight. (2016) 1:e80920. 10.1172/jci.insight.80920 27525311 PMC4982472

[101] Mattapally S Zhu W Fast VG Gao L Worley C Kannappan R. Spheroids of cardiomyocytes derived from human-induced pluripotent stem cells improve recovery from myocardial injury in mice. Am J Physiol Heart Circ Physiol. (2018) 315:H327–39. 10.1152/ajpheart.00688.2017 29631371 PMC6139622

[102] LaBarge W Mattappally S Kannappan R Fast VG Pretorius D Berry JL. Maturation of three-dimensional, hiPSC-derived cardiomyocyte spheroids utilizing cyclic, uniaxial stretch and electrical stimulation. PLoS ONE. (2019) 14:e0219442. 10.1371/journal.pone.0219442 31276558 PMC6611624

[103] Ong CS Fukunishi T Nashed A Blazeski A Zhang H Hardy S. Creation of cardiac tissue exhibiting mechanical integration of spheroids using 3D bioprinting. J Vis Exp. (2017). 10.3791/55438 28715377 PMC5608529

[104] Ong CS Fukunishi T Zhang H Huang CY Nashed A Blazeski A. Biomaterial-free three-dimensional bioprinting of cardiac tissue using human induced pluripotent stem cell derived cardiomyocytes. Sci Rep. (2017) 7:4566. 10.1038/s41598-017-05018-4 28676704 PMC5496874

[105] Zhu Y Serpooshan V Wu S Demirci U Chen P Güven S. Tissue engineering of 3D organotypic microtissues by acoustic assembly. Methods Mol Biol. (2019) 1576:301–12. 10.1007/7651_2017_68 28921421 PMC7179046

[106] Lee S Serpooshan V Tong X Venkatraman S Lee M Lee J. Contractile force generation by 3D hiPSC-derived cardiac tissues is enhanced by rapid establishment of cellular interconnection in matrix with muscle-mimicking stiffness. Biomaterials. (2017) 131:111–20. 10.1016/j.biomaterials.2017.03.039 28384492 PMC5558787

[107] Serpooshan V Chen P Wu H Lee S Sharma A Hu DA. Bioacoustic-enabled patterning of human iPSC-derived cardiomyocytes into 3D cardiac tissue. Biomaterials. (2017) 131:47–57. 10.1016/j.biomaterials.2017.03.037 28376365 PMC5446052

[108] Haraguchi Y Shimizu T Yamato M Okano T. Regenerative therapies using cell sheet-based tissue engineering for cardiac disease. Cardiol Res Pract. (2011) 2011:845170. 10.4061/2011/845170 22007333 PMC3189561

[109] Okano T Yamada N Sakai H Sakurai Y. A novel recovery system for cultured cells using plasma-treated polystyrene dishes grafted with poly(N-isopropylacrylamide). J Biomed Mater Res. (1993) 27:1243–51. 10.1002/jbm.820271005 8245039

[110] Shimizu T Yamato M Isoi Y Akutsu T Setomaru T Abe K. Fabrication of pulsatile cardiac tissue grafts using a novel 3-dimensional cell sheet manipulation technique and temperature-responsive cell culture surfaces. Circ Res. (2002) 90:e40. 10.1161/hh0302.105722 11861428

[111] Haraguchi Y Shimizu T Yamato M Kikuchi A Okano T. Electrical coupling of cardiomyocyte sheets occurs rapidly via functional gap junction formation. Biomaterials. (2006) 27:4765–74. 10.1016/j.biomaterials.2006.04.034 16737736

[112] Kreutziger KL Muskheli V Johnson P Braun K Wight TN Murry CE. Developing vasculature and stroma in engineered human myocardium. Tissue Eng Part A. (2011) 17:1219–28. 10.1089/ten.tea.2010.0557 21187004 PMC3079173

[113] Shudo Y Miyagawa S Fukushima S Saito A Shimizu T Okano T. Novel regenerative therapy using cell-sheet covered with omentum flap delivers a huge number of cells in a porcine myocardial infarction model. J Thorac Cardiovasc Surg. (2011) 142:1188–96. 10.1016/j.jtcvs.2011.07.002 21924436

[114] Kawamura M Miyagawa S Miki K Saito A Fukushima S Higuchi T. Feasibility, safety, and therapeutic efficacy of human induced pluripotent stem cell-derived cardiomyocyte sheets in a porcine ischemic cardiomyopathy model. Circulation. (2012) 126(11 Suppl. 1):S29–37. 10.1161/CIRCULATIONAHA.111.084343 22965990

[115] Kawamura M Miyagawa S Fukushima S Saito A Miki K Ito E. Enhanced survival of transplanted human induced pluripotent stem cell-derived cardiomyocytes by the combination of cell sheets with the pedicled omental flap technique in a porcine heart. Circulation. (2013) 128(11 Suppl. 1):S87–94. 10.1161/CIRCULATIONAHA.112.000366 24030425

[116] Kawamura M Miyagawa S Fukushima S Saito A Miki K Funakoshi S. Enhanced therapeutic effects of human iPS cell derived-cardiomyocyte by combined cell-sheets with omental flap technique in porcine ischemic cardiomyopathy model. Sci Rep. (2017) 7:8824. 10.1038/s41598-017-08869-z 28821761 PMC5562896

[117] Masumoto H Matsuo T Yamamizu K Uosaki H Narazaki G Katayama S. Pluripotent stem cell-engineered cell sheets reassembled with defined cardiovascular populations ameliorate reduction in infarct heart function through cardiomyocyte-mediated neovascularization. Stem Cells. (2012) 30:1196–205. 10.1002/stem.1089 22438013

[118] Sawa Y Yoshikawa Y Toda K Fukushima S Yamazaki K Ono M. Safety and efficacy of autologous skeletal myoblast sheets (TCD-51073) for the treatment of severe chronic heart failure due to ischemic heart disease. Circ J. (2015) 79:991–9. 10.1253/circj.CJ-15-0243 25912561

[119] Yoshikawa Y Miyagawa S Toda K Saito A Sakata Y Sawa Y. Myocardial regenerative therapy using a scaffold-free skeletal-muscle-derived cell sheet in patients with dilated cardiomyopathy even under a left ventricular assist device: a safety and feasibility study. Surg Today. (2018) 48:200–10. 10.1007/s00595-017-1571-1 28821963

[120] Cyranoski D ‘Reprogrammed’ stem cells approved to mend human hearts for the first time. Nature. (2018). 557:619–620. 10.1038/d41586-018-05278-8 29844563

[121] Zhang J. Engineered tissue patch for cardiac cell therapy. Curr Treat Options Cardiovasc Med. (2015). 17:399. 10.1007/s11936-015-0399-5 26122908 PMC4676725

[122] Rienks M Papageorgiou AP Frangogiannis NG Heymans S. Myocardial extracellular matrix: an ever-changing and diverse entity. Circ Res. (2014) 114:872–88. 10.1161/CIRCRESAHA.114.302533 24577967

[123] Nielsen SH Mouton AJ DeLeon-Pennell KY Genovese F Karsdal M Lindsey ML. Understanding cardiac extracellular matrix remodeling to develop biomarkers of myocardial infarction outcomes. Matrix Biol. (2019) 75-76:43–57. 10.1016/j.matbio.2017.12.001 PMC6002886 29247693

[124] Theocharis AD Skandalis SS Gialeli C Karamanos NK. Extracellular matrix structure. Adv Drug Deliv Rev. (2016) 97:4–27. 10.1016/j.addr.2015.11.001 26562801

[125] Leor J Landa N Cohen S. Renovation of the injured heart with myocardial tissue engineering. Expert Rev Cardiovasc Ther. (2006) 4:239–52. 10.1586/14779072.4.2.239 16509819

[126] Reis LA Chiu LL Feric N Fu L Radisic M. Biomaterials in myocardial tissue engineering. J Tissue Eng Regen Med. (2016) 10:11–28. 10.1002/term.1944 25066525 PMC4933503

[127] Chen QZ Harding SE Ali NN Lyon AR Boccaccini AR. Biomaterials in cardiac tissue engineering: ten years of research survey. Mater Sci Eng R Rep. (2008) 59:1–37. 10.1016/j.mser.2007.08.001

[128] Serpooshan V Mahmoudi M Hu DA Hu JB Wu SM. Bioengineering cardiac constructs using 3D printing. J 3D Print Med. (2017) 1:123–39. 10.2217/3dp-2016-0009

[129] Fukuhara S Tomita S Nakatani T Fujisato T Ohtsu Y Ishida M. Bone marrow cell-seeded biodegradable polymeric scaffold enhances angiogenesis and improves function of the infarcted heart. Circ J. (2005) 69:850–7. 10.1253/circj.69.850 15988112

[130] Engelmayr GC Jr. Cheng M Bettinger CJ Borenstein JT Langer R. Accordion-like honeycombs for tissue engineering of cardiac anisotropy. Nat Mater. (2008) 7:1003–10. 10.1038/nmat2316 18978786 PMC2613200

[131] Prabhakaran MP Kai D Ghasemi-Mobarakeh L Ramakrishna S. Electrospun biocomposite nanofibrous patch for cardiac tissue engineering. Biomed Mater. (2011) 6:055001. 10.1088/1748-6041/6/5/055001 21813957

[132] Liu BH Yeh HY Lin YC Wang MH Chen DC Lee BH. Spheroid formation and enhanced cardiomyogenic potential of adipose-derived stem cells grown on chitosan. Biores Open Access. (2013) 2:28–39. 10.1089/biores.2012.0285 23514754 PMC3569958

[133] Liu Q Tian S Zhao C Chen X Lei I Wang Z. Porous nanofibrous poly(L-lactic acid) scaffolds supporting cardiovascular progenitor cells for cardiac tissue engineering. Acta Biomater. (2015) 26:105–14. 10.1016/j.actbio.2015.08.017 26283164 PMC4584192

[134] Sidorov VY Samson PC Sidorova TN Davidson JM Lim CC Wikswo JP. I-wire heart-on-a-Chip I: three-dimensional cardiac tissue constructs for physiology and pharmacology. Acta Biomater. (2017) 48:68–78. 10.1016/j.actbio.2016.11.009 27818308 PMC5235983

[135] Hajipour MJ Mehrani M Abbasi SH Amin A Kassaian SE Garbern JC. Nanoscale technologies for prevention and treatment of heart failure: challenges and opportunities. Chem Rev. (2019) 119:11352–90. 10.1021/acs.chemrev.8b00323 31490059 PMC7003249

[136] Khan OF Sefton MV. Endothelialized biomaterials for tissue engineering applications *in vivo*. Trends Biotechnol. (2011) 29:379–87. 10.1016/j.tibtech.2011.03.004 21549438 PMC3140588

[137] Sánchez PL Fernández-Santos ME Costanza S Climent AM Moscoso I Gonzalez-Nicolas MA. Acellular human heart matrix: a critical step toward whole heart grafts. Biomaterials. (2015) 61:279–89. 10.1016/j.biomaterials.2015.04.056 26005766

[138] Ott HC Matthiesen TS Goh SK Black LD Kren SM Netoff TI. Perfusion-decellularized matrix: using nature's platform to engineer a bioartificial heart. Nat Med. (2008) 14:213–21. 10.1038/nm1684 18193059

[139] Shah M Kc P Zhang G. *In vivo* assessment of decellularized porcine myocardial slice as an acellular cardiac patch. ACS Appl Mater Interfaces. (2019) 11:23893–900. 10.1021/acsami.9b06453 31188555 PMC6948015

[140] Jia W Gungor-Ozkerim PS Zhang YS Yue K Zhu K Liu W. Direct 3D bioprinting of perfusable vascular constructs using a blend bioink. Biomaterials. (2016) 106:58–68. 10.1016/j.biomaterials.2016.07.038 27552316 PMC5300870

[141] Datta P Ayan B Ozbolat IT. Bioprinting for vascular and vascularized tissue biofabrication. Acta Biomater. (2017) 51:1–20. 10.1016/j.actbio.2017.01.035 28087487

[142] Hu JB Hu DA Buikema JW Chirikian O Venkatraman S Serpooshan V. Bioengineering of vascular myocardial tissue; a 3D bioprinting approach. Tissue Engineering Part A. (2017) 23:S158–9.

[143] Serpooshan V Hu JB Chirikian O Hu DA Mahmoudi M Wu SM. Chapter 8-4D printing of actuating cardiac tissue. In:. Min J Mosadegh B Dunham S Al'Aref S editors. 3D Printing Applications in Cardiovascular Medicine. (2018) Boston, MA (2018). p. 153–62. 10.1016/B978-0-12-803917-5.00008-0

[144] Gaetani R Doevendans PA Metz CH Alblas J Messina E Giacomello A. Cardiac tissue engineering using tissue printing technology and human cardiac progenitor cells. Biomaterials. (2012) 33:1782–90. 10.1016/j.biomaterials.2011.11.003 22136718

[145] Gaetani R Feyen DA Verhage V Slaats R Messina E Christman KL. Epicardial application of cardiac progenitor cells in a 3D-printed gelatin/hyaluronic acid patch preserves cardiac function after myocardial infarction. Biomaterials. (2015) 61:339–48. 10.1016/j.biomaterials.2015.05.005 26043062

[146] LaBarge W Morales A Pretorius D Kahn-Krell AM Kannappan R Zhang J. Scaffold-Free bioprinter utilizing layer-by-layer printing of cellular spheroids. Micromachines. (2019) 10:570. 10.3390/mi10090570 31470604 PMC6780220

[147] Ajeti V Lien CH Chen SJ Su PJ Squirrell JM Molinarolo KH. Image-inspired 3D multiphoton excited fabrication of extracellular matrix structures by modulated raster scanning. Opt Express. (2013) 21:25346–5. 10.1364/OE.21.025346 24150376

[148] Kupfer ME Lin WH Ravikumar V Qiu K Wang L Gao L. *In situ* expansion, differentiation and electromechanical coupling of human cardiac muscle in a 3D bioprinted, chambered organoid. Circ Res. (2020). 10.1161/CIRCRESAHA.119.316155 32228120 PMC8210857

[149] Marbán E. The secret life of exosomes: what bees can teach Us about next-generation therapeutics. J Am Coll Cardiol. (2018). 71:193–200. 10.1016/j.jacc.2017.11.013 29325643 PMC5769161

[150] Merino-González C Zuñiga FA Escudero C Ormazabal V Reyes C Nova-Lamperti E. Mesenchymal stem cell-derived extracellular vesicles promote angiogenesis: potencial clinical application. Front Physiol. (2016) 7:24. 10.3389/fphys.2016.00024 26903875 PMC4746282

[151] Bar A Cohen S. Inducing endogenous cardiac regeneration: can biomaterials connect the dots? Front Bioeng Biotechnol. (2020) 8:126. 10.3389/fbioe.2020.00126 32175315 PMC7056668

[152] Huang K Ozpinar EW Su T Tang J Shen D Qiao L. An off-the-shelf artificial cardiac patch improves cardiac repair after myocardial infarction in rats and pigs. Sci Transl Med. (2020) 12:eaat9683. 10.1126/scitranslmed.aat9683 32269164 PMC7293901

[153] Wang LL Liu Y Chung JJ Wang T Gaffey AC Lu M. Local and sustained miRNA delivery from an injectable hydrogel promotes cardiomyocyte proliferation and functional regeneration after ischemic injury. Nat Biomed Eng. (2017) 1:983–92. 10.1038/s41551-017-0157-y 29354322 PMC5773070

[154] Liu B Lee BW Nakanishi K Villasante A Williamson R Metz J. Cardiac recovery via extended cell-free delivery of extracellular vesicles secreted by cardiomyocytes derived from induced pluripotent stem cells. Nat Biomed Eng. (2018) 2:293–303. 10.1038/s41551-018-0229-7 30271672 PMC6159913

[155] Kolesky DB Truby RL Gladman AS Busbee TA Homan KA Lewis JA. 3D bioprinting of vascularized, heterogeneous cell-laden tissue constructs. Adv Mater. (2014). 26:3124–30. 10.1002/adma.201305506 24550124

[156] Miller JS Stevens KR Yang MT Baker BM Nguyen DH Cohen DM. Rapid casting of patterned vascular networks for perfusable engineered three-dimensional tissues. Nat Mater. (2012) 11:768–4. 10.1038/nmat3357 22751181 PMC3586565

[157] Fleischer S Shapira A Feiner R Dvir T. Modular assembly of thick multifunctional cardiac patches. Proc Natl Acad Sci USA. (2017) 114:1898–903. 10.1073/pnas.1615728114 28167795 PMC5338434

[158] Jackman CP Carlson AL Bursac N. Dynamic culture yields engineered myocardium with near-adult functional output. Biomaterials. (2016) 111:66–79. 10.1016/j.biomaterials.2016.09.024 27723557 PMC5074846

[159] Kannan RY Salacinski HJ Sales K Butler P Seifalian AM. The roles of tissue engineering and vascularisation in the development of micro-vascular networks: a review. Biomaterials. (2005) 26:1857–75. 10.1016/j.biomaterials.2004.07.006 15576160

[160] Park JH Chung BG Lee WG Kim J Brigham MD Shim J. Microporous cell-laden hydrogels for engineered tissue constructs. Biotechnol Bioeng. (2010) 106:138–48. 10.1002/bit.22667 20091766 PMC2847036

[161] Levenberg S Rouwkema J Macdonald M Garfein ES Kohane DS Darland DC. Engineering vascularized skeletal muscle tissue. Nat Biotechnol. (2005) 23:879–84. 10.1038/nbt1109 15965465

[162] Oduk Y Zhu W Kannappan R Zhao M Borovjagin AV Oparil S. VEGF nanoparticles repair the heart after myocardial infarction. Am J Physiol Heart Circ Physiol. (2018) 314:H278–84. 10.1152/ajpheart.00471.2017 29101176 PMC5867653

[163] Fan C Tang Y Zhao M Lou X Pretorius D Menasche P. CHIR99021 and fibroblast growth factor 1 enhance the regenerative potency of human cardiac muscle patch after myocardial infarction in mice. J Mol Cell Cardiol. (2020) 141:1–0. 10.1016/j.yjmcc.2020.03.003 32169551 PMC7304478

[164] Kim JJ Hou L Huang NF. Vascularization of three-dimensional engineered tissues for regenerative medicine applications. Acta Biomater. (2016) 41:17–26. 10.1016/j.actbio.2016.06.001 27262741 PMC4969172

[165] Shin SR Aghaei-Ghareh-Bolagh B Gao X Nikkhah M Jung SM Dolatshahi-Pirouz A. Layer-by-layer assembly of 3D tissue constructs with functionalized graphene. Advanced functional materials. (2014) 24:6136–44. 10.1002/adfm.201401300 25419209 PMC4235968

[166] Zhang B Montgomery M Davenport-Huyer L Korolj A Radisic M. Platform technology for scalable assembly of instantaneously functional mosaic tissues. Sci Adv. (2015) 1:e1500423. 10.1126/sciadv.1500423 26601234 PMC4643798

[167] Yang J Yamato M Kohno C Nishimoto A Sekine H Fukai F. Cell sheet engineering: recreating tissues without biodegradable scaffolds. Biomaterials. (2005) 26:6415–22. 10.1016/j.biomaterials.2005.04.061 16011847

[168] Haraguchi Y Shimizu T Sasagawa T Sekine H Sakaguchi K Kikuchi T. Fabrication of functional three-dimensional tissues by stacking cell sheets *in vitro*. Nat Protoc. (2012) 7:850–8. 10.1038/nprot.2012.027 22481530

[169] Sekine H Shimizu T Sakaguchi K Dobashi I Wada M Yamato M. *In vitro* fabrication of functional three-dimensional tissues with perfusable blood vessels. Nat Commun. (2013) 4:1399. 10.1038/ncomms2406 23360990 PMC3660653

[170] Radisic M Yang L Boublik J Cohen RJ Langer R Freed LE. Medium perfusion enables engineering of compact and contractile cardiac tissue. Am J Physiol Heart Circ Physiol. (2004) 286:H507–16. 10.1152/ajpheart.00171.2003 14551059

[171] Radisic M Deen W Langer R Vunjak-Novakovic G. Mathematical model of oxygen distribution in engineered cardiac tissue with parallel channel array perfused with culture medium containing oxygen carriers. Am J Physiol Heart Circ Physiol. (2005) 288:H1278–89. 10.1152/ajpheart.00787.2004 15539422

[172] Radisic M Park H Chen F Salazar-Lazzaro JE Wang Y Dennis R. Biomimetic approach to cardiac tissue engineering: oxygen carriers and channeled scaffolds. Tissue Eng. (2006) 12:2077–91. 10.1089/ten.2006.12.2077 16968150

[173] Vukadinovic-Nikolic Z Andrée B Dorfman SE Pflaum M Horvath T Lux M. Generation of bioartificial heart tissue by combining a three-dimensional gel-based cardiac construct with decellularized small intestinal submucosa. Tissue Eng Part A. (2014) 20:799–809. 10.1089/ten.tea.2013.0184 24102409

[174] Zhang B Montgomery M Chamberlain MD Ogawa S Korolj A Pahnke A. Biodegradable scaffold with built-in vasculature for organ-on-a-chip engineering and direct surgical anastomosis. Nat Mater. (2016) 15:669–78. 10.1038/nmat4570 26950595 PMC4879054

[175] Brown MA Iyer RK Radisic M. Pulsatile perfusion bioreactor for cardiac tissue engineering. Biotechnol Prog. (2008) 24:907–20. 10.1002/btpr.11 19194900

[176] Golden AP Tien J. Fabrication of microfluidic hydrogels using molded gelatin as a sacrificial element. Lab Chip. (2007) 7:720–5. 10.1039/b618409j 17538713

[177] Chiu LL Montgomery M Liang Y Liu H Radisic M. Perfusable branching microvessel bed for vascularization of engineered tissues. Proc Natl Acad Sci USA. (2012) 109:E3414–23. 10.1073/pnas.1210580109 23184971 PMC3528595

[178] Ye X Lu L Kolewe ME Park H Larson BL Kim ES. A biodegradable microvessel scaffold as a framework to enable vascular support of engineered tissues. Biomaterials. (2013) 34:10007–15. 10.1016/j.biomaterials.2013.09.039 24079890 PMC3899884

[179] Chaturvedi RR Stevens KR Solorzano RD Schwartz RE Eyckmans J Baranski JD. Patterning vascular networks *in vivo* for tissue engineering applications. Tissue Eng Part C Methods. (2015) 21:509–17. 10.1089/ten.tec.2014.0258 25390971 PMC4410304

[180] Cui X Boland T. Human microvasculature fabrication using thermal inkjet printing technology. Biomaterials. (2009) 30:6221–7. 10.1016/j.biomaterials.2009.07.056 19695697

[181] Kolesky DB Homan KA Skylar-Scott MA Lewis JA. Three-dimensional bioprinting of thick vascularized tissues. Proc Natl Acad Sci U S A. (2016) 113:3179–84. 10.1073/pnas.1521342113 26951646 PMC4812707

[182] Aguado BA Mulyasasmita W Su J Lampe KJ Heilshorn SC. Improving viability of stem cells during syringe needle flow through the design of hydrogel cell carriers. Tissue Eng Part A. (2012) 18:806–15. 10.1089/ten.tea.2011.0391 22011213 PMC3313609

[183] Tang XL Nakamura S Li Q Wysoczynski M Gumpert AM Wu WJ. Repeated Administrations of Cardiac Progenitor Cells Are Superior to a Single Administration of an Equivalent Cumulative Dose. J Am Heart Assoc. (2018) 7(4). 10.1161/JAHA.117.007400 29440036 PMC5850187

[184] Desgres M Menasché P. Clinical Translation of Pluripotent Stem Cell Therapies: Challenges and Considerations. Cell Stem Cell. (2019) 25:594–606. 10.1016/j.stem.2019.10.001 31703770

[185] Hare JM Traverse JH Henry TD Dib N Strumpf RK Schulman SP Reisman MA Schaer GL Sherman W. A randomized, double-blind, placebo-controlled, dose-escalation study of intravenous adult human mesenchymal stem cells (prochymal) after acute myocardial infarction. J Am Coll Cardiol. (2009) 54:2277–86. 10.1016/j.jacc.2009.06.055 19958962 PMC3580848

[186] Garcia JR Campbell PF Kumar G Langberg JJ Cesar L Wang L. A Minimally Invasive, Translational Method to Deliver Hydrogels to the Heart Through the Pericardial Space. JACC Basic Transl Sci. (2017) 2:601–609. 10.1016/j.jacbts.2017.06.003 30062173 PMC6058920

[187] Pena B Laughter M Jett S Rowland TJ M.Taylor RG Mestroni L. Injectable Hydrogels for Cardiac Tissue Engineering. Macromol Biosci. (2018) 18:e1800079. 10.1002/mabi.201800079 29733514 PMC6166441

[188] Wu T Cui C Huang Y Liu Y Fan C Han X. Coadministration of an Adhesive Conductive Hydrogel Patch and an Injectable Hydrogel to Treat Myocardial Infarction. ACS Appl Mater Interfaces. (2020) 12:2039–2048. 10.1021/acsami.9b17907 31859471

[189] Frey N Linke A Suselbeck T Muller-Ehmsen J Vermeersch P Schoors D. Intracoronary delivery of injectable bioabsorbable scaffold (IK-5001) to treat left ventricular remodeling after ST-elevation myocardial infarction: a first-in-man study. Circ Cardiovasc Interv. (2014) 7:806–12. 10.1161/CIRCINTERVENTIONS.114.001478 25351198

[190] Anker SD Coats AJ Cristian G Dragomir D Pusineri E Piredda M. A prospective comparison of alginate-hydrogel with standard medical therapy to determine impact on functional capacity and clinical outcomes in patients with advanced heart failure (AUGMENT-HF trial). Eur Heart J. (2015) 36:2297–309. 10.1093/eurheartj/ehv259 26082085 PMC4561351

[191] Gil CJ Tomov ML Theus AS Cetnar A Mahmoudi M Serpooshan V. *In vivo* tracking of tissue engineered constructs. Micromachines. (2019) 10:474. 10.3390/mi10070474 31315207 PMC6680880

[192] Mahmoudi M Zhao M Matsuura Y Laurent S Yang PC Bernstein D. Infection-resistant MRI-visible scaffolds for tissue engineering applications. Bioimpacts. (2016) 6:111–5. 10.15171/bi.2016.16 27525229 PMC4981249

[193] Shiba Y Fernandes S Zhu WZ Filice D Muskheli V Kim J. Human ES-cell-derived cardiomyocytes electrically couple and suppress arrhythmias in injured hearts. Nature. (2012) 489:322–5. 10.1038/nature11317 22864415 PMC3443324

[194] Qin X Riegler J Tiburcy M Zhao X Chour T Ndoye B. Magnetic resonance imaging of cardiac strain pattern following transplantation of human tissue engineered heart muscles. Circ Cardiovasc Imaging. (2016) 9:e004731. 10.1161/CIRCIMAGING.116.004731 27903535 PMC5378466

[195] Gao L Wang L Wei Y Krishnamurthy P Walcott GP Menasché P. Exosomes secreted by hiPSC-derived cardiac cells improve recovery from myocardial infarction in swine. Sci Transl Med. (2020) 12:eaay1318. 10.1126/scitranslmed.aay1318 32938792

[196] Shiba Y Gomibuchi T Seto T Wada Y Ichimura H Tanaka Y. Allogeneic transplantation of iPS cell-derived cardiomyocytes regenerates primate hearts. Nature. (2016) 538:388–91. 10.1038/nature19815 27723741

[197] Rai V Sharma P Agrawal S Agrawal DK. Relevance of mouse models of cardiac fibrosis and hypertrophy in cardiac research. Mol Cell Biochem. (2017) 424:123–45. 10.1007/s11010-016-2849-0 27766529 PMC5219849

[198] Romagnuolo R Masoudpour H Porta-Sánchez A Qiang B Barry J Laskary A. Human embryonic stem cell-derived cardiomyocytes regenerate the infarcted pig heart but induce ventricular tachyarrhythmias. Stem Cell Rep. (2019) 12:967–81. 10.1016/j.stemcr.2019.04.005 31056479 PMC6524945

[199] Wei K Serpooshan V Hurtado C Diez-Cunado M Zhao M Maruyama S. Epicardial FSTL1 reconstitution regenerates the adult mammalian heart. Nature. (2015) 525:479–85. 10.1038/nature15372 26375005 PMC4762253

[200] Serpooshan V Zhao M Metzler SA Wei K Shah PB Wang A. The effect of bioengineered acellular collagen patch on cardiac remodeling and ventricular function post myocardial infarction. Biomaterials. (2013) 34:9048–55. 10.1016/j.biomaterials.2013.08.017 23992980 PMC3809823

[201] Masumoto H Nakane T Tinney JP Yuan F Ye F Kowalski WJ. The myocardial regenerative potential of three-dimensional engineered cardiac tissues composed of multiple human iPS cell-derived cardiovascular cell lineages. Sci Rep. (2016) 6:29933. 10.1038/srep29933 27435115 PMC4951692

[202] Zimmermann WH. Translating myocardial remuscularization. Circ Res. (2017). 120:278–81. 10.1161/CIRCRESAHA.116.310194 28104769 PMC5312660

[203] Zhu W Zhao M Mattapally S Chen S Zhang J. CCND2 overexpression enhances the regenerative potency of human induced pluripotent stem cell–derived cardiomyocytes: remuscularization of injured ventricle. Circ Res. (2018) 122:88–96. 10.1161/CIRCRESAHA.117.311504 29018036 PMC5756126

[204] Ren J Xu Q Chen X Li W Guo K Zhao Y. Superaligned carbon nanotubes guide oriented cell growth and promote electrophysiological homogeneity for synthetic cardiac tissues. Adv Mater. (2017) 29:1702713. 10.1002/adma.201702713 29024059

[205] Wang L Wu Y Hu T Guo B Ma PX. Electrospun conductive nanofibrous scaffolds for engineering cardiac tissue and 3D bioactuators. Acta Biomater. (2017) 59:68–81. 10.1016/j.actbio.2017.06.036 28663141

[206] Gnecchi M Zhang Z Ni A Dzau VJ. Paracrine mechanisms in adult stem cell signaling and therapy. Circ Res. (2008) 103:1204–19. 10.1161/CIRCRESAHA.108.176826 19028920 PMC2667788

[207] Zimmermann WH. Remuscularization of the failing heart. J Physiol. (2017). 595:3685–90. 10.1113/JP273098 28295371 PMC5471510

[208] Malat G Culkin C. The ABCs of immunosuppression: a primer for primary care physicians. Med Clin North Am. (2016) 100:505–18. 10.1016/j.mcna.2016.01.003 27095642

[209] Pan Y Leveson-Gower DB de Almeida PE Pierini A Baker J Florek M. Engraftment of embryonic stem cells and differentiated progeny by host conditioning with total lymphoid irradiation and regulatory T cells. Cell Rep. (2015) 10:1793–802. 10.1016/j.celrep.2015.02.050 25801020 PMC4494893

[210] Braza MS M.van Leent MT Lameijer M Sanchez-Gaytan BL Arts RJW Pérez-Medina C. Inhibiting inflammation with myeloid cell-specific nanobiologics promotes organ transplant acceptance. Immunity. (2018) 49:819–28.e6. 10.1016/j.immuni.2018.09.008 30413362 PMC6251711

[211] Hsu PD Lander ES Zhang F. Development and applications of CRISPR-Cas9 for genome engineering. Cell. (2014) 157:1262–78. 10.1016/j.cell.2014.05.010 24906146 PMC4343198

[212] Kim H Kim JS. A guide to genome engineering with programmable nucleases. Nat Rev Genet. (2014) 15:321–4. 10.1038/nrg3686 24690881

[213] Mattapally S Pawlik KM Fast VG Zumaquero E Lund FE Randall TD. Human leukocyte antigen class I and II knockout human induced pluripotent stem cell–derived cells: universal donor for cell therapy. J Am Heart Assoc. (2018) 7:e010239. 10.1161/JAHA.118.010239 30488760 PMC6405542

[214] Deuse T Hu X Gravina A Wang D Tediashvili G De C. Hypoimmunogenic derivatives of induced pluripotent stem cells evade immune rejection in fully immunocompetent allogeneic recipients. Nat Biotechnol. (2019) 37:252. 10.1038/s41587-019-0016-3 30778232 PMC6419516

[215] Xu H Wang B Ono M Kagita A Fujii K Sasakawa N. Targeted disruption of HLA genes via CRISPR-Cas9 generates iPSCs with enhanced immune compatibility. Cell Stem Cell. (2019) 24:566–78. e7. 10.1016/j.stem.2019.02.005 30853558

